# Interplay between astrocyte reactivity and APOE ε4 status is associated with accelerated pTau-related tau pathology in Alzheimer’s disease

**DOI:** 10.1186/s13024-025-00906-0

**Published:** 2025-10-29

**Authors:** Xiaoxie Mao, Yan Wang, Ying Luan, Ying Wang, Jie Wang, Wenlin Dai, Yihui Guan, Qi Huang, Roger N. Gunn, Rik Ossenkoppele, Binyin Li, Zijing Li, Qihao Guo, Fang Xie

**Affiliations:** 1 Department of Nuclear Medicine & PET Center, Huashan Hospital, Fudan University, Shanghai, China; 2 Center for Molecular Imaging and Translational Medicine, State Key Laboratory of Molecular Vaccinology and Molecular Diagnostics, School of Public Health, Xiamen University, Xiamen, Fujian 361102 China; 3 School of Medicine, Xiamen University, Xiamen, Fujian 361102 China; 4 Department of Radiology, School of Medicine, Zhongda Hospital, Southeast University, Nanjing, China; 5 Department of Gerontology, Shanghai Sixth People’s Hospital, Shanghai Jiao Tong University School of Medicine, 600 Yishan Road, Shanghai, 200233 China; 6 Center for Applied Statistics, Institute of Statistics and Big Data, Renmin University of China, Beijing, China; 7 Department of Brain Sciences, Imperial College London, London, UK; 8 Clinical Memory Research Unit, Department of Clinical Sciences in Malmö, Lund University, Lund, Sweden; 9 Alzheimer Center Amsterdam, Neurology, Vrije Universiteit Amsterdam, Amsterdam UMC Location VUmc, Amsterdam, The Netherlands; 10 Amsterdam Neuroscience, Neurodegeneration, Amsterdam, The Netherlands; 11 Department of Neurology and Institute of Neurology, Ruijin Hospital, Shanghai Jiao Tong University School of Medicine, Shanghai, China; 12 MOE Frontiers Center for Brain Science, Fudan University, Shanghai, China

**Keywords:** Alzheimer’s disease, Phosphorylated tau, Glial fibrillary acidic protein, APOE ε4, Tau PET

## Abstract

**Background:**

Various plasma phosphorylated tau species have been shown to be associated with amyloid-β (Aβ) PET and Tau PET in Alzheimer’s disease (AD), but whether APOE ε4 affects the interaction between glial fibrillary acidic protein (GFAP) and phosphorylated tau (pTau), and whether a three-way interaction exists among APOE ε4, GFAP, and pTau that influences AD progression remain unclear.

**Methods:**

The study included 563 participants from the Chinese Preclinical Alzheimer’s Disease Study (CPAS) and 243 from Alzheimer’s Disease Neuroimaging Initiative (ADNI), all of whom underwent Aβ PET, magnetic resonance imaging (MRI), neuropsychological assessments, and plasma biomarker analyses (GFAP, pTau181, pTau231, pTau217), with subsets undergoing Tau PET. The longitudinal data of 101 participants from ADNI were additionally included. We employed linear regression models with interaction terms to examine how APOE ε4 status and plasma GFAP levels modulate the relationships between plasma pTau biomarkers and AD pathology cross-sectionally and longitudinally.

**Results:**

Plasma GFAP and pTau biomarkers (pTau181, pTau231, pTau217) are significantly elevated in Aβ-positive individuals, with stronger Aβ–pTau associations observed in APOE ε4 carriers (CPAS: β = 0.26, *p* = 0.003 for pTau231; ADNI: β = 0.45, *p* < 0.001 for pTau181). Across two cohorts, plasma GFAP levels significantly strengthened the associations between pTau biomarkers and Tau PET. Furthermore, subsequent analyses revealed that this modulatory effect of GFAP on the links between pTau and PET-derived pathological changes was more pronounced in APOE ε4 non-carriers, whereas in APOE ε4 carriers, a significant interaction between GFAP and pTau was only observed in specific Braak stage-specific regions within the CPAS cohort. In longitudinal analyses, we also observed stronger pTau181-associated longitudinal tau accumulation in individuals with high GFAP levels (Braak III-IV).

**Conclusion:**

We demonstrate that APOE ε4 status critically modulates the relationship between pTau and Aβ pathology, whereas plasma GFAP primarily influences pTau–tau pathology associations, particularly in individuals without APOE ε4 allele. These findings underscore the role of reactive astrogliosis in tau propagation and support the utility of plasma biomarkers for AD diagnosis and prognosis.

**Supplementary Information:**

The online version contains supplementary material available at 10.1186/s13024-025-00906-0.

## Introduction

Alzheimer’s disease (AD) is the most common neurodegenerative disorder and a leading cause of dementia worldwide, imposing a significant public health and economic burden. It is pathologically characterized by the deposition of amyloid-β (Aβ) plaques, tau neurofibrillary tangles, and a prominent neuroinflammatory response. Early diagnosis of AD is particularly challenging due to symptom overlap with normal aging and the need for invasive or expensive diagnostic tools such as cerebrospinal fluid (CSF) analysis and positron emission tomography (PET) imaging [1, 2]. Among genetic risk factors, the APOE ε4 genotype is the most strongly associated with sporadic AD, influencing amyloid and tau pathology as well as neuroinflammatory responses, which are closely interlinked in disease progression [3–5]. Understanding these complex interactions is critical for developing minimally invasive, accessible biomarkers to improve diagnosis and monitoring across the AD continuum.

Plasma biomarkers including glial fibrillary acidic protein (GFAP) and phosphorylated tau species (pTau181, pTau231, pTau217) now enable non-invasive assessment of AD pathology and neuroinflammation [6]. Ultra-sensitive immunoassays have made large-scale biomarker studies feasible [7, 8]. Multiple studies have consistently confirmed that different p-tau isoforms are closely associated with brain Aβ and tau pathology, with their dynamic changes in plasma levels accurately reflecting the complex relationship between amyloid pathology and tau deposition [9–13]. Beyond classical pathological protein alterations, neuroinflammation also plays an increasingly important role in the AD pathological cascade. GFAP, as a specific marker of astrocyte activation, shows significantly elevated plasma levels in AD patients [14]. While early studies suggested that GFAP primarily reflects Aβ-related pathological changes, recent evidence indicates that GFAP also participates in tau aggregation and cortical thinning processes [14, 15].

APOE ε4, as the strongest genetic risk factor for sporadic AD, has pathogenic mechanisms far more complex than simply promoting Aβ deposition. APOE ε4 carriers exhibit enhanced Aβ-related tau spreading, an effect that can be monitored through longitudinal changes in plasma pTau217 [16]. In APOE ε4 carriers, pTau more readily progresses to brain regions affected by Aβ [17]. More importantly, selective removal of APOE ε4 from astrocytes significantly reduces tau-mediated neurodegeneration, a finding that emphasizes the central role of astrocyte-derived APOE ε4 in AD pathological progression [18]. Interestingly, APOE genotype produces differential effects on the associations between biomarkers and pathological changes. A study of clinically unimpaired elderly individuals found that the correlation between plasma pTau and Aβ-PET was mainly observed in APOE ε3/3 carriers, while the correlation between plasma GFAP and Aβ-PET primarily appeared in APOE ε4/4 carriers [19]. This genotype-specific difference suggests that APOE genotype may modulate the interaction between neuroinflammation and tau pathology through different mechanisms.

Although previous studies have provided important insights into AD pathological mechanisms, several critical knowledge gaps remain unresolved. Based on existing evidence, we hypothesized that additional molecular interactions may contribute to disease progression but have not been fully elucidated. Therefore, in this study, we utilized two independent cohorts and applied a comprehensive approach combining cross-sectional and longitudinal analyses with both plasma biomarkers and PET imaging to specifically address the following key questions: (1) whether APOE ε4 genotype and plasma GFAP modulate the relationship between plasma pTau and AD pathologies, (2) whether the interaction among APOE ε4, GFAP, and pTau differentially influences AD pathologies. Elucidating these interactions may deepen our understanding of AD pathophysiology and contribute to the development of more precise diagnostic and therapeutic strategies.

## Methods

### Participants

The study involved a cohort of 563 participants from the Chinese Preclinical Alzheimer’s Disease Study (CPAS), including 365 cognitively impaired (CI) patients and 198 cognitively unimpaired (CU) individuals. Participants were recruited from Huashan Hospital, Fudan University, and Ruijin Hospital, affiliated with Shanghai Jiao Tong University School of Medicine. The CI group consisted of 160 patients with mild cognitive impairment (MCI) and 205 patients with dementia. All participants underwent Aβ PET ([^18^F]florbetapir [FBP]) and magnetic resonance imaging (MRI) scans, provided blood samples for plasma biomarker analyses (GFAP, pTau181: *n* = 530; pTau231: *n* = 442; pTau217: *n* = 419), and completed a comprehensive set of neuropsychological assessments. A subset of 432 participants (282 with CI and 150 with CU) also underwent Tau PET ([^18^F]MK6240) scans. The study protocol adhered to ethical guidelines and was approved by the Institutional Review Boards of Huashan Hospital, Fudan University, and Ruijin Hospital. Written informed consent was obtained from all participants prior to their enrollment.

An independent sample of 243 participants was included from the Alzheimer’s Disease Neuroimaging Initiative (ADNI) database (http://ida.loni.usc.edu), comprising 145 CU individuals and 98 CI patients. A detailed listing of the specific ADNI files used for data extraction is provided in the Supplementary Material. All included participants underwent Aβ PET (FBP), Tau PET ([^18^F]flortaucipir [FTP]), and MRI, as well as a comprehensive battery of neuropsychological assessments. Plasma biomarker data were available in subsets of the cohort (GFAP: *n* = 222; pTau181: *n* = 224; pTau217: *n* = 197). Of these, 101 participants had longitudinal follow-up data for both Aβ PET and Tau PET, though 21 participants had missing Aβ PET data. Data were accessed and used in accordance with the ADNI Data Use Agreement. The ADNI was launched in 2003 as a public-private partnership, led by Principal Investigator Michael W. Weiner, MD. The study protocol was approved by the institutional review boards of all participating sites, and written informed consent was obtained from each participant or their legally authorized representative.

In the CPAS cohort, Aβ-positive status was determined through qualitative visual assessment [20–22]. In the ADNI cohort, Aβ positivity was defined based on global SUVR thresholds, with a cutoff of ≥ 1.11 for FBP [23]. On the basis of these results, the subjects were categorized into amyloid PET-positive (A+) and PET-negative (A−) groups.

### Neuropsychological assessment

In the CPAS cohort, a comprehensive neuropsychological test battery encompassing six key tests across three cognitive domains, along with the Mini-Mental State Examination (MMSE), a global cognitive test, was conducted. Memory was evaluated via the Auditory Verbal Learning Test (AVLT), which includes 30-minute delayed free recall (AVLT-LDR, 12 items) and a recognition component (24 items). The language assessment included the Animal Fluency Test (AFT) total score and the Boston Naming Test (BNT) 30-item total score. Attention and executive function were gauged via the Shape Trail Test (STT) Parts A and B.

The diagnostic criteria for AD and MCI were established on the basis of the 2018 National Institute on Aging-Alzheimer’s Association (NIA-AA) criteria for AD dementia and a modified version of Jak and Bondi’s criteria [24–26], respectively. MCI was diagnosed when participants exhibited impairment in one or more cognitive domains or impaired scores (>1 SD) in each of the three cognitive domains [27]. Participants without any evidence of cognitive impairment were classified as CU.

In the ADNI cohort, memory performance was evaluated using the ADNI-MEM composite score, which integrates multiple memory assessments, while global cognitive status was measured with the MMSE [28]. Participants were classified as CU if they had an MMSE score ≥ 24, a Clinical Dementia Rating (CDR) of 0, and no signs of depression; those with a CDR ≥ 0.5 were considered CI [29, 30].

### Measurement of blood-based biomarkers

In the CPAS cohort, plasma GFAP levels were quantified using the Quanterix Neurology 4-Plex E (N4PE) assay. Plasma pTau181, pTau231, and pTau217 levels were measured with commercial immunoassays from Quanterix (pTau-181 V2 Advantage Kit and pTau-231 Advantage Plus Kit) and ALZpath (pTau-217 v2) [31–33].

In the ADNI cohort, plasma GFAP was quantified using the Quanterix Neurology 4-Plex E assay on the Simoa HD-X platform. Plasma pTau181 was measured using the Roche Elecsys NeuroToolKit platform, and pTau217 was assessed with the ALZpath assay on the Quanterix Simoa HD-X system at the Quanterix Accelerator Laboratory. Detailed methodological protocols are available through the ADNI database (adni.loni.usc.edu).

### PET and MR imaging

In the CPAS cohort, [^18^F]florbetapir (Aβ) and [^18^F]MK6240 (Tau) PET imaging, along with MRI, were performed as previously reported [22, 34]. High-resolution 3-dimensional (3D) MR images were acquired using 3T MR scanners (Prisma, Siemens, Germany). The 3D magnetization-prepared rapid gradient-echo sequence was employed to obtain MRI T1-weighted images with the following parameters: repetition time = 3000 ms, echo time = 2.56 ms, flip angle = 7°, acquisition matrix = 320 × 320, in-plane resolution = 0.8 mm × 0.8 mm, and slice thickness = 0.8 mm, resulting in a total of 208 sagittal slices. PET/CT imaging using [^18^F]MK6240 and [^18^F]florbetapir was carried out on a PET/CT scanner (Biograph-mCT Flow, Siemens, Erlangen, Germany) with well-established parameters, as previously described [35, 36]. Scans lasting 20 min were performed at 50 min post injection of ~ 370 MBq (± 10%) [^18^F]florbetapir and 90 min post injection of ~ 185 MBq (± 10%) [^18^F]MK6240. Following data acquisition, the PET images were reconstructed via a filtered back-projection algorithm, which incorporates corrections for attenuation, normalization, dead time, photon attenuation, scatter, and random coincidences. The final PET image matrix had dimensions of 168 × 168 × 148, with each voxel set to 2.04 × 2.04 × 1.50 mm^3^. In the ADNI cohort, structural MRI was performed on 3T scanners (Siemens, GE, and Philips) using MPRAGE T1-weighted sequences (TR = 2300ms, 1 mm³ isotropic voxels). PET imaging utilized [^18^F]florbetapir for amyloid (4 × 5 min frames, 50–70 min post-injection) and [^18^F]flortaucipir for tau (6 × 5 min frames, 75–105 min post-injection). Detailed protocols are available at http://adni.loni.usc.edu/methods/pet-analysis-method/pet-analysis/.

### Image preprocessing

Extensive information on PET data processing procedures for the ADNI cohort can be found on the official ADNI website (http://adni-info.org). In the CPAS cohort, image preprocessing was performed using SPM12 (Wellcome Trust Centre for Neuroimaging, London, UK; https://www.fil.ion.ucl.ac.uk/spm) following a previously reported method [35]. First, the PET and T1-weighted MR images were reoriented, and the PET images were subsequently coregistered to the corresponding T1-weighted images. The T1-weighted images were segmented into gray matter (GM), white matter (WM), and CSF, and then normalized to the standard Montreal Neurological Institute (MNI) space. The resulting normalization parameters were then applied to the corresponding PET images. The normalized PET was smoothed via a Gaussian filter with a full width at half maximum (FWHM) of 8 mm. For volume-of-interest (VOI) analysis, the PET images underwent spatial normalization and intensity normalization without smoothing.

Brain region values for [^18^F]florbetapir were extracted, and standardized uptake value ratios (SUVR) were calculated using Centiloid standard mask images (voi_cxt_2mm.nii and voi_WhlCb_2mm.nii) provided by the Global Alzheimer’s Association Interactive Network (GAAIN).

To ensure consistency with GAAIN preprocessing results, we validated the approach using the standard PIB dataset, which also provided a conversion formula for transforming [^11^C]PIB SUVR into Centiloids.

$$\eqalign{Centiloid\_PIB & = 100 \times {{SUVR\_PIB - 1.009} \over {2.074 - 1.009}} \cr& = 100 \times {{SUVR\_PIB - 1.009} \over {1.065}} \cr} $$

In the Florbetapir Calibration dataset, a linear regression model was developed to define the relationship between [^18^F]florbetapir and [^11^C]PIB SUVR using direct comparisons of their images.

$$\:y=0.523x+0.513$$

We then derived the conversion between [^18^F]Florbetapir and [^11^C]PIB SUVR.

$$\:SUVR\_Florbetapir=0.523\times\:SUVR\_PIB+0.513$$

$$\:SUVR\_PIB=\frac{SUVR\_Florbetapir-0.513}{0.523}$$

In summary, we established a formula for directly calculating Centiloid from [^18^F]Florbetapir SUVR.

$$\eqalign{Centiloids = & 179.64 \times SUVR\_Florbetapir - 186.95 \cr} $$

For [^18^F]MK6240 PET imaging, SUVR was calculated using the inferior cerebellar gray matter as the reference region [22, 34]. To assess regional tau deposition, SUVR was further quantified for three composite regions corresponding to Braak stages I-II (entorhinal cortex and hippocampus), III-IV (limbic regions including parahippocampal and temporal areas), and V-VI (neocortical regions) [22, 37].

### Statistical analysis

All statistical analyses were conducted using R software (version 4.3.1) and SPM12. The normality assumption was assessed using the Shapiro‒Wilk test. As the concentrations of blood biomarkers were not normally distributed, natural logarithmic (ln) transformations were applied for subsequent analyses. Continuous variables were summarized as mean ± standard deviation (SD), and categorical variables were reported as frequencies and percentages. Group comparisons were performed using one-way ANOVA for continuous variables and the Chi-squared test for categorical variables. Statistical significance was defined as a *p*-value < 0.05 unless stated otherwise.

One-way analysis of covariance (ANCOVA) was performed to compare plasma biomarker levels among the four groups (Aβ− CU, Aβ+ CU, Aβ− CI, and Aβ+ CI), with age and sex as covariates. Post hoc pairwise comparisons were conducted with Bonferroni correction for multiple comparisons. Cohen’s d values were calculated to estimate the effect sizes for these comparisons. Similarly, participants were stratified by APOE ε4 carrier status (non-carriers vs. carriers) into CU-nonε4, CU-ε4, CI-nonε4, and CI-ε4 groups, and the same statistical methods were applied to these comparisons.

To investigate the relationships between baseline plasma biomarkers and AD pathology, linear regression models were used to evaluate associations between plasma biomarkers and two key pathological markers: amyloid-β burden and tau protein deposition. Models were adjusted for age, sex, years of education, and diagnostic group (CU vs. CI). Additionally, voxel-wise correlation analyses were conducted to explore the relationships between baseline plasma biomarkers and AD pathology using multiple linear regression (MLR) models in SPM12. These analyses were adjusted for age, sex, years of education, and diagnostic group, with significance set at an FDR-corrected threshold of *p* < 0.05. When stratified by cognitive status (CU and CI), models were adjusted for age, sex, and years of education only.

To examine whether APOE ε4 status and plasma GFAP levels modulate the associations between plasma pTau biomarkers and AD pathology, we constructed linear regression models incorporating interaction terms (e.g., pTau × APOE ε4 status, pTau × GFAP). To explore regional specificity, we conducted ROI-based analyses using the 200 Schaefer parcels. The same interaction terms (e.g., pTau × APOE status, pTau × GFAP) were tested in each ROI with FDR correction (*p* < 0.05). All models were adjusted for age, sex, years of education, and clinical diagnosis. For analyses with Tau PET as the dependent variable, we examined associations both with and without adjustment for Centiloid to assess the independent effects. GFAP was modeled as a continuous variable, while stratification by median (low vs. high) was applied for visualization purposes. To further explore potential effect modification by APOE ε4 status, subgroup analyses were conducted within APOE ε4 carrier and non-carrier groups.

For longitudinal analyses in the ADNI cohort, where most participants had only two to three Tau PET scans, individual slopes of Aβ-PET and Tau PET SUVR change were estimated using linear regression in participants with at least two timepoints. The same covariate structure and standardization procedures were applied. Interaction terms (e.g., pTau × APOE status, pTau × GFAP) were used to assess whether these factors jointly influenced the rates of Aβ and tau accumulation over time.

## Results

### Sample characteristics

Table 1 summarizes the demographic and clinical characteristics at baseline and during follow-up, stratified by cognitive status and Aβ positivity. The CPAS cohort comprised 563 participants (CU−, *n* = 166; CU+, *n* = 32; CI−, *n* = 142; CI+, *n* = 223). The mean age was 69.0 years (SD = 8.22), with 60.6% female participants. All participants underwent Aβ PET imaging. Significant group differences were observed in age, years of education, APOE ε4 carrier status, amyloid and tau burden, and cognitive performance ( *p* < 0.001).

**Table 1:** Demographic information

	CPAS					ADNI			
	All (*N* = 563)	CU- (*N* = 166)	CU+ (*N* = 32)	CI- (*N* = 142)	CI+ (*N* = 223)	All (*N* = 243)	CU- (*N* = 84)	CU+ (*N* = 61)	CI- (*N* = 51)
**Age**									
Mean (SD)	69.0 (8.22) ***	65.7 (7.76) c**	67.8 (8.21)	69.2 (7.78)b**	71.3 (8.19)	77.2 (7.25) **	76.6 (6.83)	80.0 (6.54) a*	75.4 (7.03) d**
**Sex (%)**									
Male	222 (39.4%)	68 (41.0%)	12 (37.5%)	55 (38.7%)	87 (39.0%)	127 (52.3%) *	40 (47.6%)	24 (39.3%)	31 (60.8%)
Female	341 (60.6%)	98 (59.0%)	20 (62.5%)	87 (61.3%)	136 (61.0%)	116 (47.7%)	44 (52.4%)	37 (60.7%)	20 (39.2%)
**Education**,** year**									
Mean (SD)	11.7 (3.77) ***	13.1 (3.54) c***	13.2 (2.87)	10.8 (3.70)b***, d**	11.0 (3.84) e*	16.6 (2.61)	16.9 (2.43)	16.6 (2.44)	16.7 (3.01)
**APOE ε4 (%)**									
non-carrier	349 (62.0%) ***	132 (79.5%) c***	19 (59.4%)	104 (73.2%)	94 (42.2%)f***	163 (67.1%) ***	66 (78.6%)	37 (60.7%)	41 (80.4%)
carrier	205 (36.4%)	32 (19.3%)	13 (40.6%)	36 (25.4%)	124 (55.6%)	80 (32.9%)	18 (21.4%)	24 (39.3%)	10 (19.6%)
Missing	9 (1.6%)	2 (1.2%)	0 (0%)	2 (1.4%)	5 (2.2%)				
**Centiloids**									
Mean (SD)	24.8 (36.1) ***	0.0682 (15.9) c***	37.1 (30.5)a***	0.933 (20.6)d***	56.5 (29.2) e**, f***	30.8 (44.3) ***	-0.952 (10.1) c***	66.3 (38.4) a***	-2.22 (12.3) d***
**Braak I II (SUVR)**									
Mean (SD)	1.18 (0.436) ***	0.849 (0.0962) c***	1.08 (0.310) a***	0.957 (0.330) d*	1.53 (0.377) e***,f***	1.19 (0.221) ***	1.10 (0.123) c***	1.21 (0.171) a***	1.12 (0.184) d**
Missing	131 (23.3%)	43 (25.7%)	6 (18.8%)	53 (35.3%)	44 (19.1%)				
**Braak III IV (SUVR)**									
Mean (SD)	1.38 (0.648) ***	0.957 (0.0749) c***	1.10 (0.222) a**	1.05 (0.325)	1.87 (0.695) e***,f***	1.17 (0.173) ***	1.11 (0.0869) c***	1.17 (0.128)	1.12 (0.0962)
Missing	131 (23.3%)	43 (25.7%)	6 (18.8%)	53 (35.3%)	44 (19.1%)				
**Braak V VI (SUVR)**									
Mean (SD)	1.22 (0.569) ***	0.926 (0.0753) c***	0.994 (0.156)	0.948 (0.207)	1.59 (0.699) e***,f***	1.07 (0.144) ***	1.03 (0.0797) c***	1.07 (0.106)	1.03 (0.0824)
Missing	131 (23.3%)	43 (25.7%)	6 (18.8%)	53 (35.3%)	44 (19.1%)				
**MMSE**									
Mean (SD)	23.6 (5.97) ***	28.0 (1.61) c***	27.4 (1.54)	23.7 (5.39) b***, d***	19.4 (6.12),e***,f***	28.0 (3.07) ***	29.1 (1.21) c***	28.2 (1.95) a*	28.8 (1.65)
Missing						1 (0.4%)	1 (1.2%)	0 (0%)	0 (0%)
**Participants with longitudinal Aβ PET and Tau PET**									
**Sample size**									
**Tau PET**						**101**	**31**	**27**	**26**
**Aβ PET**						**21 (20.8%)**	**5 (16.1%)**	**7 (25.9%)**	**3 (11.5%)**
**Participant’s number of Aβ PET scans**									
Mean (SD)						2.29 (0.532)	2.31 (0.618)	2.35 (0.489)	2.30 (0.559)
**Participant’s number of Tau PET scans**									
Mean (SD)						2.53 (0.742)	2.39 (0.715)	2.74 (0.813)	2.58 (0.758)
**Duration Aβ PET**,** years**									
Mean (SD)						3.10 (1.28) *	3.46 (1.33) c*	2.83 (1.05)	3.33 (1.35)
**Duration Tau PET**,** years**									
Mean (SD)						2.76 (1.41) *	3.06 (1.52) c*	2.57 (1.18)	3.10 (1.46)

Note: a: CU- vs. CU+, b: CU- vs. CI-, c: CU- vs. CI+, d: CU+ vs. CI-, e: CU+ vs. CI+, f: CI- vs. CI+; *, **, and *** represent significant differences across groups at *p* < 0.05, 0.01, and 0.001. Values are presented as mean ± standard deviation (SD) for continuous variables and n (%) for categorical variables. *p*-values were calculated using ANOVA for continuous variables and χ² tests for categorical variables

Abbreviations: CU−, cognitively unimpaired and amyloid-negative; CU+, cognitively unimpaired and amyloid-positive; CI−, cognitively impaired and amyloid-negative; CI+, cognitively impaired and amyloid-positive; SUVR, standardized uptake value ratio; MMSE, Mini-Mental State Examination

The ADNI cohort included 243 participants (CU−, *n* = 84; CU+, *n* = 61; CI−, *n* = 51; CI+, *n* = 47), with a mean age of 77.2 years (SD = 7.25) and 47.7% females. All participants underwent both Aβ and Tau PET imaging. Significant differences across groups were also observed in age, sex, APOE ε4 status, amyloid and tau burden, and cognitive outcomes (*p* < 0.001). Additionally, we presented scatter plots comparing Aβ PET (Centiloid) and Tau PET (SUVR) values across groups to visualize the distribution of pathological burden (Supplemental Fig. 1).

A subset of 101 participants (31 CU–, 27 CU+, 26 CI–, 17 CI+) underwent longitudinal Tau PET imaging, with up to 3 scans (mean ± SD: 2.53 ± 0.742 scans) over 2.76 ± 1.41 years. Among these participants, 81 also had longitudinal Aβ PET data available, with up to 2 scans (2.29 ± 0.532 scans) over a mean follow-up period of 3.1 ± 1.28 years. In both cohorts, we further characterized the distribution of APOE genotypes across diagnostic and Aβ-defined subgroups, quantified the number and concentrations of plasma biomarkers assessed, and evaluated cognitive performance across multiple neuropsychological domains (Supplemental Table 1), and presented the demographic characteristics of participants with longitudinal follow-up (Supplemental Table 2).

Furthermore, plasma GFAP and pTau biomarkers (pTau181, pTau231, and pTau217) were elevated in Aβ-positive individuals. Additionally, in the CPAS cohort, significant increases in these biomarkers were observed across clinical stages and APOE ε4 status (Supplemental Fig. 2). Moreover, in both cohorts, plasma GFAP levels were positively correlated with pTau concentrations (Supplemental Fig. 3).

### Associations of plasma GFAP and pTau with Aβ and Tau PET

**Fig. 1:**
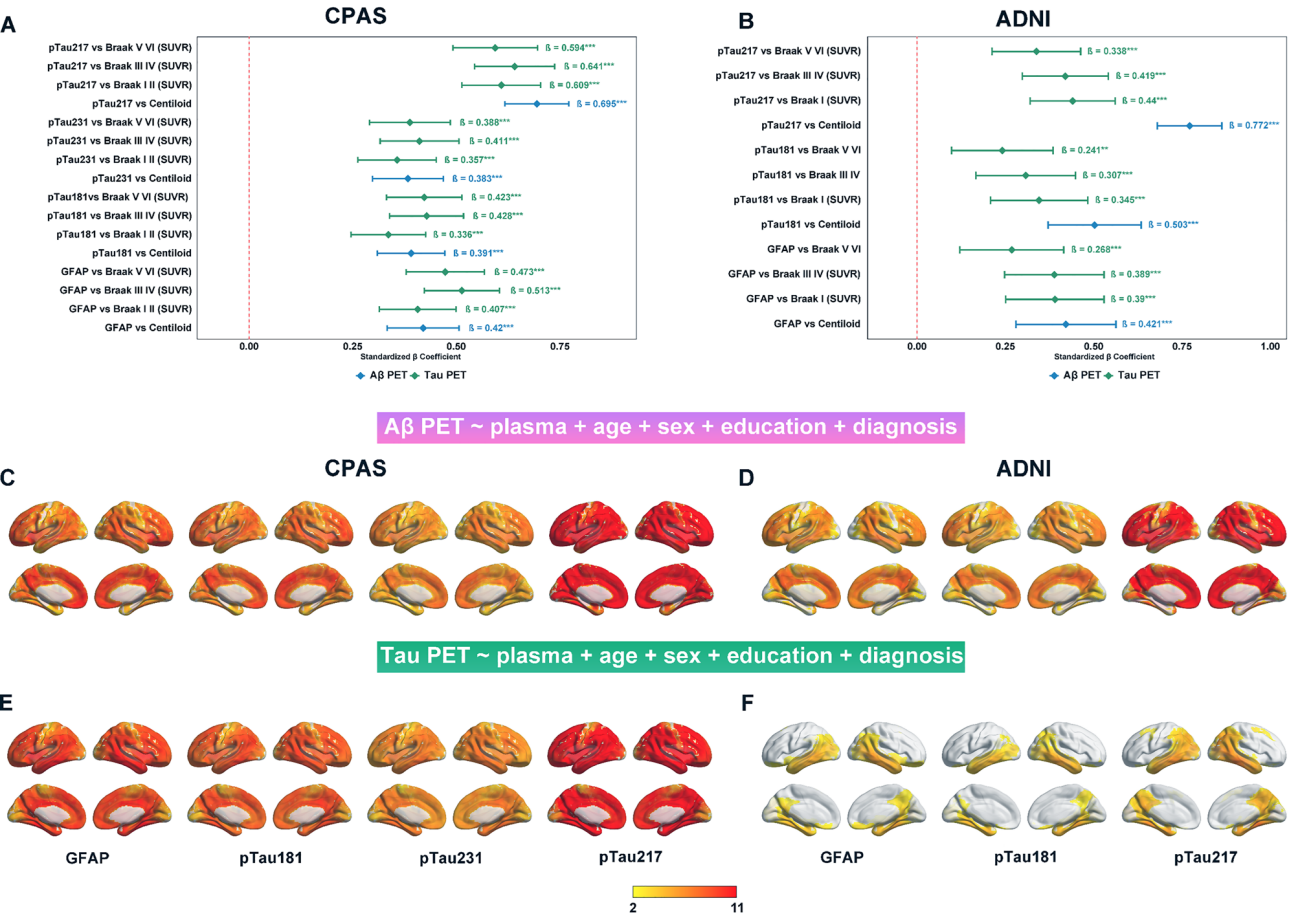
Associations of plasma GFAP and phosphorylated tau with Aβ and Tau PET. Plasma GFAP and pTau were examined for their associations with Aβ PET and Tau PET measures in the CPAS (**A**) and ADNI (**B**) cohorts using ROI analyses. Voxel-wise associations between plasma GFAP and pTau with Aβ PET were evaluated in CPAS (**C**) and ADNI (**D**), and with Tau PET in CPAS (**E**) and ADNI (**F**). Forest plots illustrate standardized β coefficients and their 95% confidence intervals from linear regression models; green indicates associations with Aβ PET and blue with Tau PET. Voxel-wise statistical maps display T values, with significance defined at *p* < 0.05 (peak-level FDR correction). All models were adjusted for age, sex, years of education and clinical diagnosis. Plasma GFAP and pTau were log-transformed prior to inclusion in the models. **p < 0.01, ***p < 0.001

In both the CPAS and ADNI cohorts, plasma levels of GFAP and phosphorylated tau (pTau181, pTau217, pTau231) were significantly associated with Aβ and Tau PET. In ROI-based analyses, plasma pTau217 showed the strongest association with global Aβ PET burden across both cohorts (CPAS: standardized β = 0.695, 95% CI: 0.62–0.77, *p* < 0.001; ADNI: standardized β = 0.772, 95% CI: 0.68–0.86, *p* < 0.001) (Fig. 1A–B).

Voxel-wise analyses further supported these findings. In CPAS, widespread cortical associations were observed between plasma GFAP and pTau and both Aβ PET (Fig. 1C) and Tau PET (Fig. 1E), with prominent effects in temporoparietal and posterior cingulate regions. Similar spatial patterns were observed in ADNI (Fig. 1D and F), although the effects were more restricted, particularly for Tau PET, where significant associations with pTau181 and GFAP were largely confined to the medial temporal lobe. Notably, pTau217 exhibited the most extensive spatial overlap with elevated Tau PET. To further explore the early and late associations between plasma biomarkers and Aβ and tau burden, we conducted voxel-based analyses stratified by CU/CI groups (Supplemental Fig. 4). We also examined the relationship between Aβ PET (Centiloid) and Tau PET (SUVR) in both cohorts, as tau analyses may be influenced by Centiloid values (Supplemental Fig. 5). Additionally, by stratifying the analysis by diagnostic groups, we assessed the associations of plasma phosphorylated tau (pTau) with Aβ and Tau PET in each diagnostic category, which provided context for our subsequent interaction analyses (Supplemental Fig. 6).

### Stronger Aβ–pTau associations in APOE ε4 carriers

**Fig. 2:**
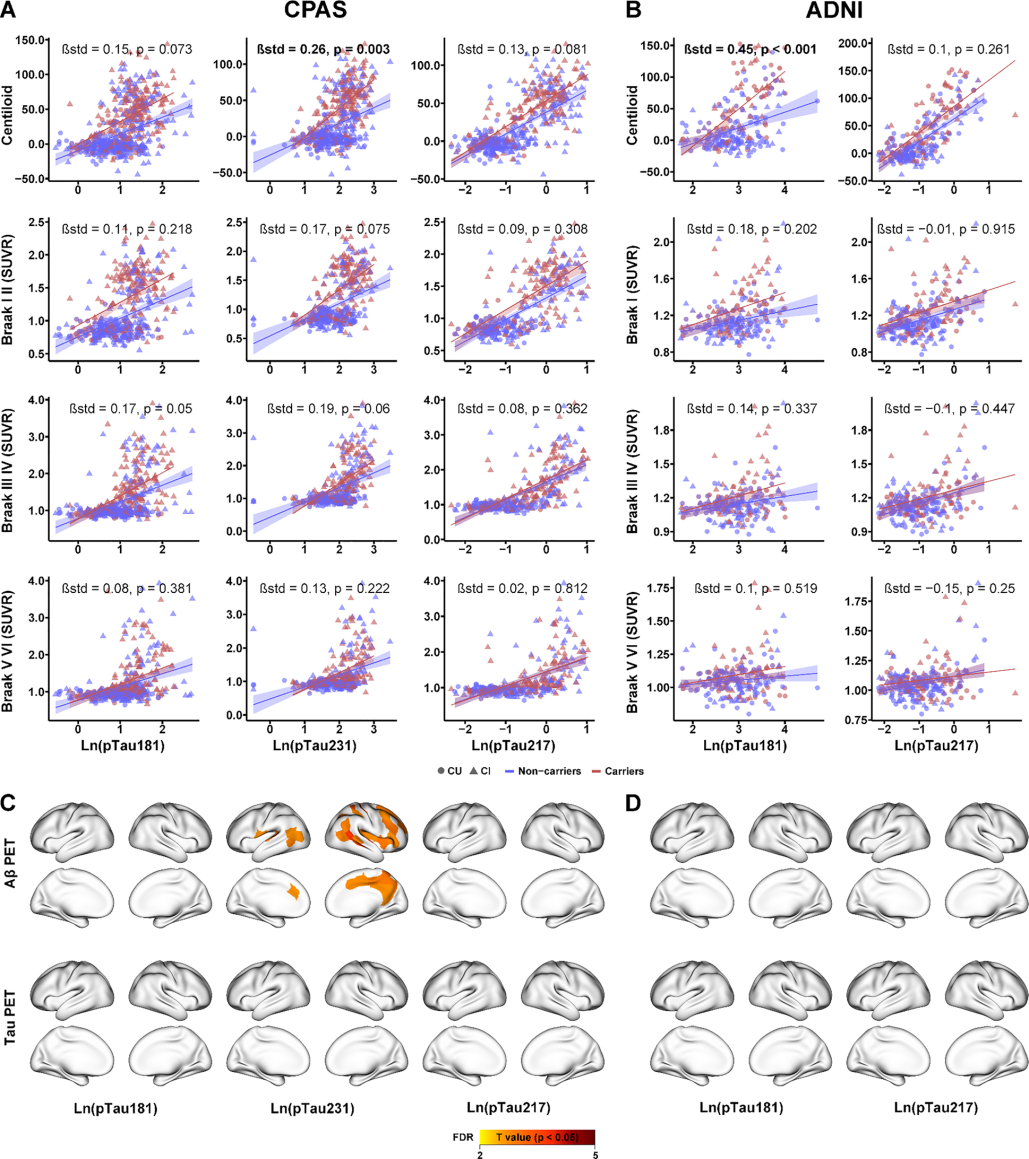
Interaction effect between plasma phosphorylated tau (pTau) and APOE ε4 status on Aβ and Tau PET. Plasma pTau and its interaction with APOE ε4 carrier status were assessed for their associations with Aβ PET and Tau PET at the ROI level in the CPAS (**A**) and ADNI (**B**) cohorts. ROI-wise interactions across 200 cortical regions (defined by the Schaefer atlas) are shown in brain maps for CPAS (**C**) and ADNI (**D**). Linear regression models are shown with 95% confidence intervals and accompanied by standardized β coefficients, along with regression lines and individual data points stratified by APOE ε4 status (red = carriers, blue = non-carriers). Circles and triangles represent CU and CI participants, respectively. Positive or negative T values reflect stronger or weaker pTau–associated Aβ or tau burden in ε4 carriers compared with non-carriers. All models were adjusted for age, sex, years of education, and clinical diagnosis. Plasma GFAP and pTau were log-transformed prior to inclusion in the models. SUVR = standardized uptake value ratio

We next examined whether APOE ε4 status modulated the associations between Aβ PET or Tau PET and plasma pTau biomarkers. In the CPAS cohort, a significant interaction between APOE ε4 status and plasma pTau231 was observed on Aβ PET (standardized β = 0.26; 95% CI, 0.09–0.44; *p* = 0.003), with spatial effects primarily localized to the right lateral somatomotor cortex, temporoparietal regions, and medial prefrontal cortex (Fig. 2A and C). In contrast, in the ADNI cohort, a significant interaction was found between APOE ε4 status and plasma pTau181 on Aβ PET (standardized β = 0.45; 95% CI, 0.19–0.70; *p* < 0.001) (Fig. 2B); however, no spatially consistent or statistically significant parcel-level effects were detected in the corresponding brain maps (Fig. 2D). Importantly, no significant interactions between APOE ε4 status and plasma pTau181, pTau231, or pTau217 were observed with Tau PET in either cohort. Additionally, we included Aβ PET (Centiloid) values as a covariate in the model, and the results remained largely unchanged (Supplemental Fig. 7).

### Higher plasma GFAP enhances the association between plasma pTau and Tau PET

**Fig. 3:**
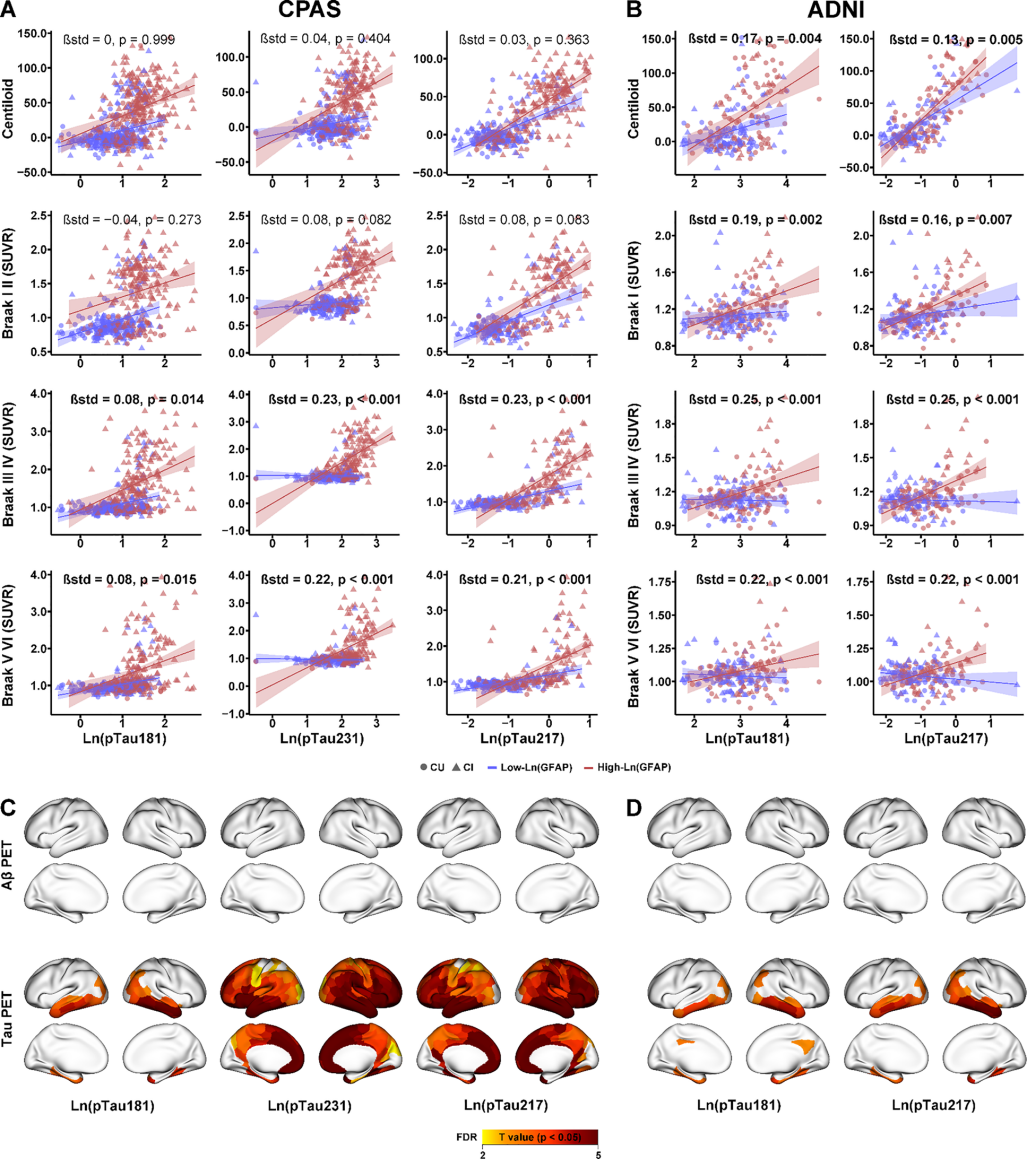
Interaction effect between plasma phosphorylated tau (pTau) and GFAP on Aβ and Tau PET. Plasma pTau and its interaction with plasma GFAP were assessed for their associations with Aβ PET and Tau PET at the ROI level in the CPAS (**A**) and ADNI (**B**) cohorts. ROI-wise interactions across 200 cortical regions (defined by the Schaefer atlas) are shown in brain maps for CPAS (**C**) and ADNI (**D**). Linear regression models are shown with 95% confidence intervals and accompanied by standardized β coefficients, along with regression lines and individual data points stratified by GFAP level (red = high GFAP, blue = low GFAP). Circles and triangles represent CU and CI participants, respectively. Positive or negative T values reflect stronger or weaker pTau–associated Aβ or tau burden in individuals with high GFAP compared with those with low GFAP. All models were adjusted for age, sex, years of education, and clinical diagnosis. Plasma GFAP and pTau were log-transformed prior to inclusion in the models. All interactions were modeled using continuous plasma GFAP values; GFAP levels were dichotomized at the median for visualization purposes. SUVR = standardized uptake value ratio

We next assessed whether plasma GFAP levels modulate the relationship between pTau biomarkers and Aβ or Tau PET. In the CPAS cohort, plasma GFAP did not significantly interact with pTau biomarkers in relation to Aβ PET (Fig. 3A). In contrast, in the ADNI cohort, higher GFAP levels significantly strengthened the associations between pTau181 (standardized β = 0.17; 95% CI, 0.06–0.28; *p* = 0.004) and pTau217 (standardized β = 0.13; 95% CI, 0.04–0.23; *p* = 0.005) and Aβ PET (Fig. 3B). Importantly, in both cohorts, plasma GFAP significantly moderated the association between pTau biomarkers and Tau PET, with stronger pTau–Tau PET relationships observed in participants with elevated GFAP levels (Fig. 3).

In the CPAS cohort, regions showing significant interaction effects differed by plasma pTau. For pTau181, significant interactions were predominantly localized to the prefrontal cortex, limbic system, and cingulate regions. pTau231 demonstrated more widespread interaction effects. In contrast, pTau217 showed significant interactions primarily in the prefrontal and temporal cortices, extending into the control and limbic networks (Fig. 3C). In the ADNI cohort, Plasma pTau181 was associated with bilateral temporal lobes, dorsal attention network, and visual cortex. Plasma pTau217 further involved the right parietal default mode network and broader visual cortex (Fig. 3D). Additionally, we included Aβ PET (Centiloid) values as a covariate in the model, and the associations with Tau PET remained significant (Supplemental Fig. 8).

### Higher plasma GFAP enhances pTau–Tau PET associations in APOE ε4 non-carriers

**Fig. 4:**
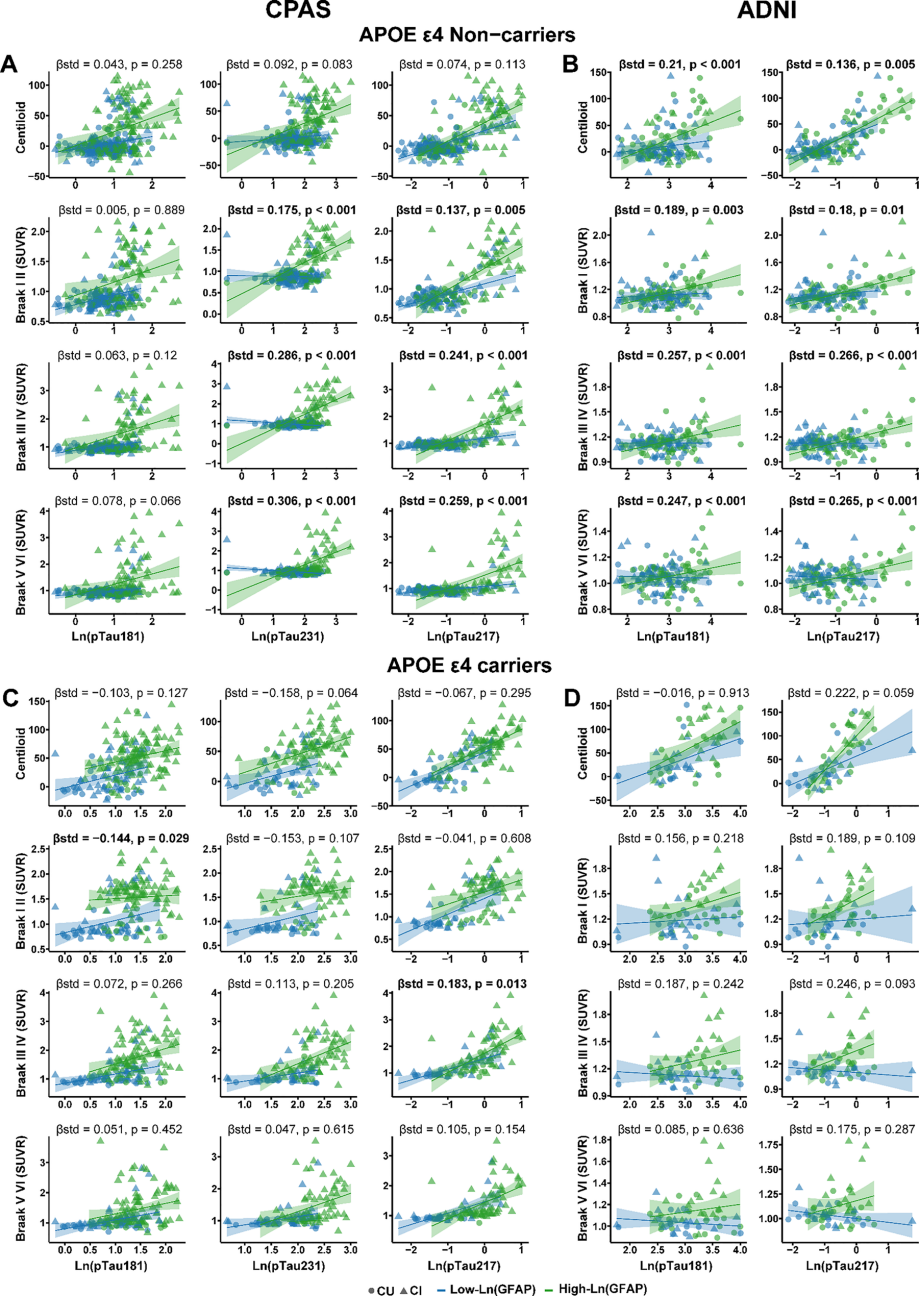
Interaction effect between plasma phosphorylated tau (pTau) and GFAP on Aβ PET and Tau PET stratified by APOE ε4 status. Associations of plasma pTau and its interaction with plasma GFAP with Aβ PET and Tau PET at the ROI level were assessed in APOE ε4 non-carriers in the CPAS (**A**) and ADNI (**B**) cohorts. Panels C and D show the corresponding associations in APOE ε4 carriers in the CPAS (**C**) and ADNI (**D**) cohorts. Linear regression models are shown with 95% confidence intervals and accompanied by standardized β coefficients, along with regression lines and individual data points stratified by GFAP level (green = high GFAP, blue = low GFAP). Circles and triangles represent CU and CI participants, respectively. All models were adjusted for age, sex, years of education, and clinical diagnosis. Plasma GFAP and pTau values were log-transformed prior to inclusion in the models. All interactions were modeled using continuous plasma GFAP values; GFAP levels were dichotomized at the median for visualization purposes. SUVR = standardized uptake value ratio

We further investigated whether the association between plasma GFAP and pTau biomarkers in relation to Aβ and Tau PET differed by APOE ε4 carrier status. In the CPAS cohort, no significant interaction was observed between plasma GFAP and pTau biomarkers on Aβ PET among APOE ε4 non-carriers. However, in the ADNI cohort, significant interactions were detected in non-carriers between plasma GFAP and both pTau181 (standardized β = 0.21; 95% CI, 0.10–0.32; *p* < 0.001) and pTau217 (standardized β = 0.14; 95% CI, 0.04–0.23; *p* = 0.005) on Aβ PET (Fig. 4A–B).

In the CPAS cohort, significant interactions between plasma GFAP and pTau231, as well as between plasma GFAP and pTau217, were observed in non-carriers with respect to Tau PET. Similarly, in the ADNI cohort, plasma GFAP significantly interacted with pTau181 and pTau217 in relation to Tau PET among non-carriers. These findings suggest that in APOE ε4 non-carriers, higher plasma GFAP levels amplify the association between pTau biomarkers and Tau PET (Fig. 4A–B).

In contrast, among APOE ε4 carriers in both cohorts, plasma GFAP did not show significant interactions with pTau biomarkers on either Aβ or Tau PET. An exception was observed in the CPAS cohort, where plasma GFAP exhibited a significant interaction with pTau181 in Braak I–II regions and with pTau217 in Braak III–IV regions (Fig. 4C–D).

### Higher plasma GFAP amplifies pTau181–related longitudinal tau accumulation

**Fig. 5:**
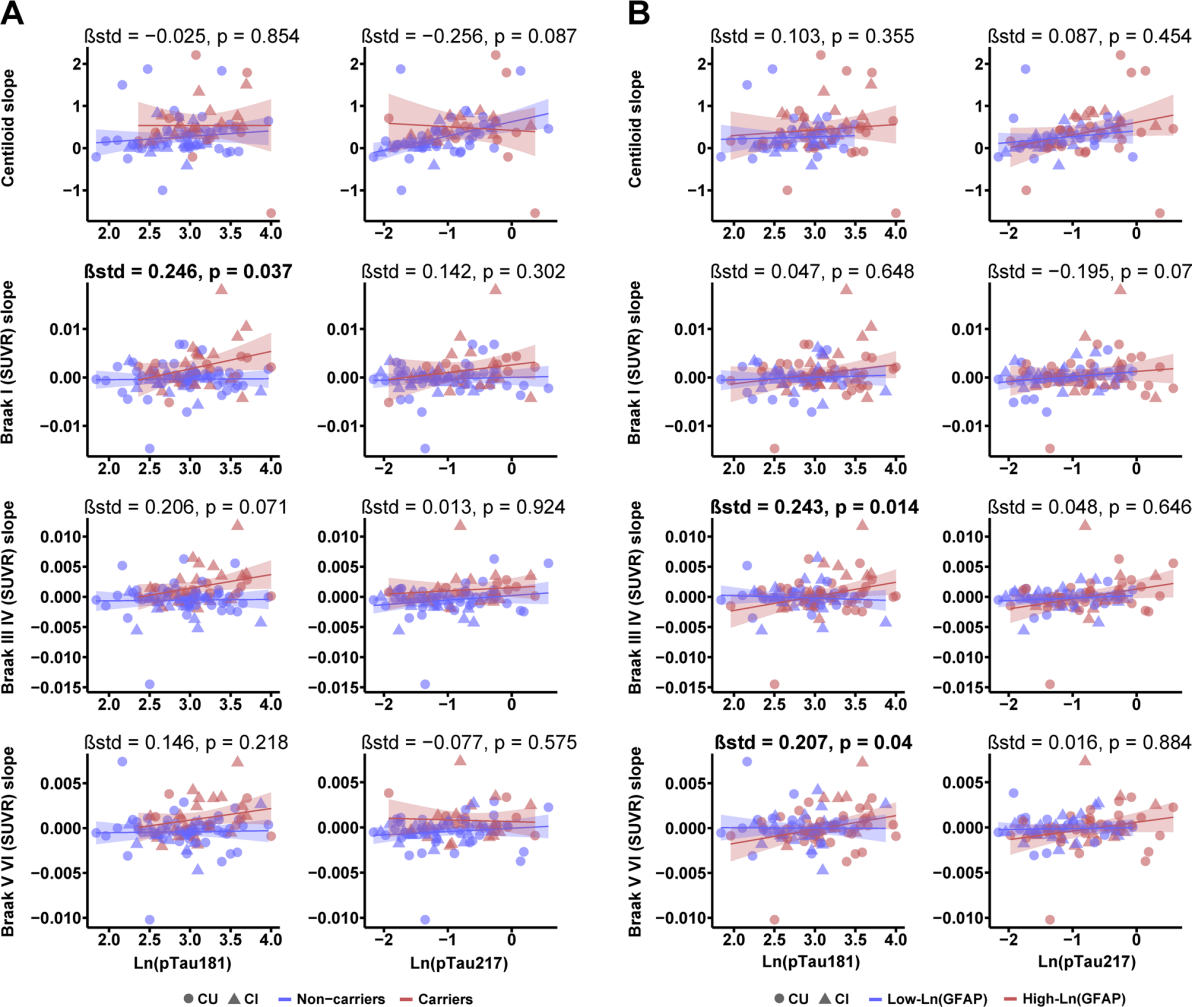
Interaction of plasma pTau (pTau181 and pTau217) with APOE ε4 status and GFAP predicts longitudinal changes in Aβ and Tau PET. Associations between plasma pTau181 and pTau217 and the annual rate of change in Aβ PET (Centiloid) and Tau PET SUVR were examined in the ADNI cohort. (**A**) shows the interaction of pTau with APOE ε4 carrier status, while (**B**) shows the interaction of pTau with plasma GFAP. In panels **A** and **B**, red/blue indicate APOE ε4 carriers or high GFAP, and non-carriers or low GFAP, respectively. Circles and triangles represent CU and CI participants, respectively. All models were adjusted for age, sex, years of education, and clinical diagnosis. Plasma GFAP and pTau values were log-transformed prior to inclusion in the models. All interactions were modeled using continuous plasma GFAP values; GFAP levels were dichotomized at the median for visualization purposes. SUVR = standardized uptake value ratio

In the ADNI cohort, among participants with longitudinal Aβ and Tau PET (*n* = 101), we further examined the interactive effects of plasma pTau, APOE ε4 status, and GFAP levels on Aβ and tau accumulation. We found that neither the interaction between plasma pTau and APOE ε4 status nor that between plasma pTau and GFAP was significantly associated with Aβ accumulation (Fig. 5A and B). However, in participants with higher plasma GFAP, pTau181 was significantly associated with faster tau accumulation, particularly in Braak III–IV regions (standardized β = 0.243; 95% CI, 0.243–0.244; *p* = 0.014) and Braak V–VI regions (standardized β = 0.207; 95% CI, 0.207–0.208; *p* = 0.04) (Fig. 5B). Interestingly, no such effect was observed for pTau217.

## Discussion

This study investigated the associations between plasma phosphorylated tau (pTau181, pTau231, and pTau217) and Aβ and tau pathology across two cohorts. Differential modulatory effects of APOE ε4 status and plasma GFAP were identified on the associations between plasma pTau biomarkers and both Aβ and tau pathology. Specifically, stronger Aβ deposition was observed in APOE ε4 carriers in association with pTau231 (CPAS) and pTau181 (ADNI) levels. Moreover, higher plasma GFAP levels were associated with enhanced tau deposition linked to pTau181, pTau231 and pTau217. Further analysis revealed that in APOE ε4 non-carriers, higher plasma GFAP levels were specifically associated with strengthened correlations between plasma pTau and tau deposition. In longitudinal analyses from the ADNI, higher plasma GFAP levels were associated with pTau181-related tau accumulation across Braak stages III–VI. In summary, carriers of the APOE ε4 allele exhibit not only greater Aβ deposition but may also exacerbate Aβ accumulation through modulation of pTau-related mechanisms. In contrast, elevated GFAP is associated with pTau-related tau deposition, with this effect being particularly prominent in APOE ε4 non-carriers. Moreover, high plasma GFAP levels further contribute to pTau181-associated longitudinal tau accumulation. These findings suggest that astrocytic activation may serve as a key non-genetic driver of tau pathology progression, rather than merely a downstream response to Aβ deposition.

Among the pTau isoforms examined, pTau217 showed the strongest associations with both Aβ and Tau PET burden in both cohorts. This observation is consistent with recent findings, suggesting that pTau217 may be the most sensitive plasma biomarker for detecting AD pathology [38–40]. Furthermore, we observed that APOE ε4 status and plasma GFAP levels differentially modulate the relationships between these pTau biomarkers and pathological changes.

A significant interaction between APOE ε4 status and plasma pTau biomarkers on Aβ PET burden indicates that genetic risk modulates the link between peripheral biomarkers and cerebral pathology. Previous studies have confirmed that APOE ε4 carriers exhibit accelerated Aβ accumulation [41, 42]. APOE ε4 impairs Aβ clearance, resulting in earlier and more extensive amyloid deposition, which may in turn trigger stronger tau phosphorylation and the release of tau into circulation. Additionally, APOE ε4 carriers display blood–brain barrier (BBB) disruption [42], potentially facilitating greater peripheral release of brain-derived proteins and amplifying biomarker–pathology associations. The spatial pattern of these enhanced associations, particularly within temporoparietal and medial prefrontal cortices, matches regions of early Aβ deposition in APOE ε4 carriers, further supporting a mechanistic link between genetic risk, regional vulnerability, and elevated biomarker levels. This gene–biomarker interaction underscores the clinical need to account for APOE genotype when interpreting plasma pTau levels, as identical biomarker concentrations may correspond to different degrees of brain pathology depending on genetic background.

Another key finding was that elevated plasma GFAP markedly strengthened the association between plasma pTau biomarkers and Tau PET. This effect was most pronounced in participants without the APOE ε4 allele. These results suggest that in the absence of strong genetic risk, neuroinflammatory processes reflected by GFAP may play a critical role in the progression of tau pathology. The spatial distribution of these effects involved the prefrontal cortex, limbic system, and primary sensory cortices, indicating that GFAP-related mechanisms may influence tau accumulation across distinct brain networks.

Notably, our findings suggest that GFAP may also act as a driver of AD pathology. Traditionally, GFAP elevation has been viewed as a reactive consequence of Aβ deposition rather than an initiator of pathological processes [14]. However, emerging perspectives propose that plasma GFAP may contribute directly to tau aggregation and cortical thinning [15]. Recent experimental data further support an active role of astrocytes; a selective reduction of astrocytic APOE expression significantly reduced Aβ plaque deposition, indicating that astrocytes are not merely responders but also key regulators within the pathological cascade [43]. In this study, the modulation of pTau–tau pathology associations by GFAP—especially evident in APOE ε4 non-carriers—supports this regulatory role. This implies that in the absence of genetically mediated inflammation, GFAP-linked neuroinflammation may emerge as a principal driver of tau pathology progression [44, 45]. As demonstrated by Montagne et al., APOE ε4 carriers exhibit BBB disruption independent of Aβ and tau pathology, mediated via the cyclophilin A–MMP9 pathway [42]. Consequently, in APOE ε4 carriers, vascular pathology may serve as the dominant pathological driver, potentially masking or replacing astrocyte-mediated inflammatory mechanisms.

Longitudinal analyses confirmed this observation. Higher plasma GFAP specifically enhanced the rate of pTau181-related tau accumulation, validating the robustness of cross-sectional findings and revealing a sustained and cumulative pathological effect driven by plasma GFAP [46]. It is hypothesized that Aβ pathology may trigger initial neuroinflammation, which facilitates tau deposition, while subsequent tau pathology may, in turn, activate astrocytic responses. This self-reinforcing pathological cascade may explain why disease progression can occur in individuals lacking genetic susceptibility. However, due to limited direct evidence, these mechanistic interpretations remain speculative and should be approached with caution. Nevertheless, our results highlight the contextual role of GFAP in the plasma pTau–tau aggregation relationship and lay the groundwork for future clinical investigations into their potential interplay. Of particular interest, this accelerating effect was observed only for pTau181, but not for pTau217. Given that pTau217 is considered a marker of earlier pathological stages [47–49], this difference may reflect stage-specific dynamics: pTau217 may capture initial pathological triggers, whereas pTau181 reflects ongoing neurodegeneration, which is more susceptible to modulation by neuroinflammation. Ultimately, neuroinflammation-driven tau propagation may serve as an alternative pathway in APOE ε4 non-carriers, offering novel therapeutic targets for this subgroup. These genotype-dependent effects underscore the need for personalized anti-inflammatory interventions based on APOE genotype—astrocyte-targeted therapies may be more effective for APOE ε4 non-carriers, whereas carriers may require combinatorial multi-target approaches.

Several limitations of this study should be acknowledged. First, insufficient longitudinal follow-up in the CPAS cohort precluded further verification of the interaction effects over time. Moreover, the limited ADNI follow-up sample (*n* = 101) prevented further analysis of how APOE ε4 status influences the GFAP modulation of pTau-associated longitudinal tau accumulation. Second, differences in cohort characteristics (e.g., ethnicity, education), biomarker assay platforms, and PET processing pipelines may have contributed to inconsistencies, particularly regarding specific pTau isoforms that showed significant interactions. Moreover, while plasma GFAP is a sensitive marker of astrocyte activation, its specificity remains limited. Elevated GFAP levels are also observed in various neurological conditions, including multiple sclerosis and cerebrovascular disease. Future studies should incorporate combinations of astrocyte-related biomarkers—such as YKL-40 and S100B—to achieve a more comprehensive characterization of astrocytic activation.

## Conclusion

This study demonstrates that APOE ε4 genotype and plasma GFAP play crucial roles in governing the associations between plasma pTau biomarkers and cerebral Aβ and tau pathology. The most salient finding is that elevated plasma GFAP significantly strengthened the association between pTau and tau pathology—particularly in APOE ε4 non-carriers—and increased the rate of pTau181-related tau accumulation. These results deepen our understanding of astrocyte reactivity’s role in tau pathology propagation and underscore the critical importance of targeting astrocyte-mediated neuroinflammation in Alzheimer’s disease.

## Supplementary Information

Below is the link to the electronic supplementary material.

Supplementary Material 1

## Data Availability

Data from the Huashan cohort are not publicly available but may be obtained from the authors upon reasonable request and with permission from the relevant ethics committees.

## Abbreviations

Aβ: Amyloid-β
AD: Alzheimer’s disease
ANCOVA: Analysis of covariance
pTau: Phosphorylated tau
GFAP: Glial fibrillary acidic protein
CSF: Cerebrospinal fluid
PET: Positron emission tomography
Simoa: Single-molecule array
MCI: Mild cognitive impairment
MRI: Magnetic resonance imaging
MMSE: Mini-Mental State Examination
AVLT: Auditory Verbal Learning Test
AFT: Animal Fluency Test
BNT: Boston Naming Test
STT: Shape Trail Test
NIA-AA: National Institute on Aging-Alzheimer’s Association
MNI: Montreal Neurological Institute
GM: Gray matter
WM: White matter
FWHM: Full width at half maximum
VOI: Volume-of-interest
SUVR: Standardized uptake value ratios
GAAIN: Global Alzheimer’s Association Interactive Network
MLR: Multivariate linear regression
BBB: Blood–brain barrier
