# Supplementary material for: Interplay between astrocyte reactivity and APOE ε4 status is associated with accelerated pTau-related tau pathology in Alzheimer’s disease
Source: Mol Neurodegener. 2025 Oct 29;20:115. doi: 10.1186/s13024-025-00906-0 (PMC12573940; doi:10.1186/s13024-025-00906-0)
Supplement: Supplementary file 1 — Supplementary Material 1 [file 13024_2025_906_MOESM1_ESM.docx]

**Supplemental materials**

**Interplay between astrocyte reactivity and APOE ε4 status is associated with accelerated pTau-related tau pathology in Alzheimer’s disease**

Xiaoxie Mao^1, 2, 3#^, Yan Wang^1#^, Ying Luan^1, 4#^, Ying Wang^5#^, Jie Wang^1^, Wenlin Dai^6^, Yihui Guan^1^, Qi Huang^1^, Roger N. Gunn^7^, Rik Ossenkoppele ^8, 9, 10^, for the Alzheimer’s Disease Neuroimaging Initiative, Binyin Li^11*^, Zijing Li^2*^, Qihao Guo^5*^, Fang Xie^1,12*^

The following **Alzheimer’s Disease Neuroimaging Initiative (ADNI)** data files were used in this study and can be found in the **Supplementary Material** section:

1. **REGISTRY**
2. **UCBERKELEY_AMY_6MM**
3. **ADNI_UCD_WMH_05_02_22**
4. **UCBERKELEY_TAU_6MM**
5. **UCBERKELEYFDG_8mm_02_17_23**
6. **UCBERKELEYAV1451_04_26_22**
7. **All_Subjects_PTDEMOG**
8. **All_Subjects_First_Visit**
9. **All_Subjects_DXSUM**
10. **All_Subjects_APOERES**
11. **All_Subjects_ADAS_ADNIGO23**
12. **All_Subjects_ADASSCORES**
13. **All_Subjects_CDR**
14. **All_Subjects_MMSE**
15. **All_Subjects_MOCA**
16. **All_Subjects_NEUROBAT**
17. **All_Subjects_FAQ**
18. **All_Subjects_NPIQ**
19. **All_Subjects_GDSCALE**
20. **All_Subjects_FNIHBC_BLOOD_BIOMARKER_TRAJECTORIES**
21. **All_Subjects_UPENNBIOMK_ROCHE_ELECSYS**
22. **BLENNOW_LAB_CSF_GAP_43_06_08_21**
23. **ADNI_HAASS_WASHU_LAB**
24. **BLENNOWCSFNFL**
25. **BLENNOWCSFNG**
26. **UCSFFSX51_11_08_19**
27. **UCSFFSX6_07_06_23**
28. **UCSFFSX51_ADNI1_3T_02_01_16**

These datasets were obtained from the ADNI database and were used for the analysis presented in this study.

**Supplemental Table 1. Demographic information.**

|  | **CPAS** |  |  |  |  |  | **ADNI** |  |  |  |  |  |  |
| --- | --- | --- | --- | --- | --- | --- | --- | --- | --- | --- | --- | --- | --- |
|  | **All (N = 563)** | **CU- (N = 166)** | **CU+ (N = 32)** | **CI- (N = 142)** | **CI+ (N = 223)** |  | **All (N = 243)** | **CU- (N = 84)** | **CU+ (N = 61)** | **CI- (N = 51)** | **CI+ (N = 47)** |  |  |
| **APOE genotype** |  |  |  |  |  |  |  |  |  |  |  |  |  |
| E2/E2 | 4 (0.7%) | 3 (1.8%) | 1 (3.1%) | 0 (0%) | 0 (0%) |  | 1 (0.4%) | 1 (1.2%) | 0 (0%) | 0 (0%) | 0 (0%) |  |  |
| E2/E3 | 50 (8.9%) | 24 (14.5%) | 1 (3.1%) | 15 (10.6%) | 10 (4.5%) |  | 24 (9.9%) | 9 (10.7%) | 6 (9.8%) | 9 (17.6%) | 0 (0%) |  |  |
| E2/E4 | 8 (1.4%) | 3 (1.8%) | 1 (3.1%) | 2 (1.4%) | 2 (0.9%) |  | 3 (1.2%) | 0 (0%) | 1 (1.6%) | 0 (0%) | 2 (4.3%) |  |  |
| E3/E3 | 295 (52.4%) | 105 (63.3%) | 17 (53.1%) | 89 (62.7%) | 84 (37.7%) |  | 138 (56.8%) | 56 (66.7%) | 31 (50.8%) | 32 (62.7%) | 19 (40.4%) |  |  |
| E3/E4 | 167 (29.7%) | 27 (16.3%) | 11 (34.4%) | 31 (21.8%) | 98 (43.9%) |  | 62 (25.5%) | 16 (19.0%) | 20 (32.8%) | 10 (19.6%) | 16 (34.0%) |  |  |
| E4/E4 | 30 (5.3%) | 2 (1.2%) | 1 (3.1%) | 3 (2.1%) | 24 (10.8%) |  | 15 (6.2%) | 2 (2.4%) | 3 (4.9%) | 0 (0%) | 10 (21.3%) |  |  |
| Missing | 9 (1.6%) | 2 (1.2%) | 0 (0%) | 2 (1.4%) | 5 (2.2%) |  |  |  |  |  |  |  |  |
| **GFAP (pg/mL)** |  |  |  |  |  |  |  |  |  |  |  |  |  |
| Mean (SD) | 92.2 (77.5) *** | 52.8 (29.5) c*** | 93.5 (66.6) a*** | 73.1 (49.8) b*** | 138 (97.1) e***,f*** |  | 181 (92.2) *** | 160 (79.9) c* | 227 (94.9) a*** | 136 (70.2) d*** | 216 (97.6) f*** |  |  |
| Missing | 33 (5.9%) | 3 (1.8%) | 1 (3.1%) | 3 (2.1%) | 26 (11.7%) |  | 34 (14.0%) | 11 (13.1%) | 9 (14.8%) | 3 (5.9%) | 11 (23.4%) |  |  |
| **pTau181 (pg/mL)** |  |  |  |  |  |  |  |  |  |  |  |  |  |
| Mean (SD) | 3.39 (1.99) *** | 2.40 (1.15) c*** | 3.30 (1.35) a*** | 2.87 (1.81) | 4.60 (2.13) e**,f*** |  | 22.4 (12.9) *** | 18.1 (8.91) c*** | 28.7 (17.0) a*** | 17.8 (8.39) d*** | 28.2 (11.9) f*** |  |  |
| Missing | 33 (5.9%) | 3 (1.8%) | 1 (3.1%) | 3 (2.1%) | 26 (11.7%) |  | 35 (14.4%) | 12 (14.3%) | 9 (14.8%) | 3 (5.9%) | 11 (23.4%) |  |  |
| **pTau231 (pg/mL)** |  |  |  |  |  |  |  |  |  |  |  |  |  |
| Mean (SD) | 7.91 (4.10) *** | 5.56 (2.59) c*** | 7.15 (3.38) | 7.03 (3.37) b*** | 10.4 (4.25) e**,f*** |  |  |  |  |  |  |  |  |
| Missing | 121 (21.5%) | 37 (22.3%) | 8 (25.0%) | 23 (16.2%) | 53 (23.8%) |  |  |  |  |  |  |  |  |
| **pTau217 (pg/mL)** |  |  |  |  |  |  |  |  |  |  |  |  |  |
| Mean (SD) | 0.699 (0.563) *** | 0.306 (0.166) c*** | 0.643 (0.317) a*** | 0.446 (0.331) b***, d** | 1.19 (0.559) e***,f*** |  | 0.532 (0.531) *** | 0.294 (0.119) c*** | 0.758 (0.475) a*** | 0.298 (0.144) d** | 0.946 (0.890) f*** |  |  |
| Missing | 144 (25.6%) | 42 (25.3%) | 9 (28.1%) | 29 (20.4%) | 64 (28.7%) |  | 10 (4.1%) | 3 (3.6%) | 1 (1.6%) | 1 (2.0%) | 5 (10.6%) |  |  |
| **AVLT_LDR** |  |  |  |  |  |  |  |  |  |  |  |  |  |
| Mean (SD) | 2.21 (2.64) *** | 4.73 (2.50) c*** | 3.52 (2.39) | 1.91 (2.08) b***, d** | 0.350 (0.977) e***,f*** |  | 10.3 (3.36) *** | 11.7 (2.29) c*** | 10.9 (3.09) | 10.0 (3.37) b* | 7.20 (3.40) e***,f*** |  |  |
| Missing |  |  |  |  |  |  | 2 (0.8%) | 1 (1.2%) | 0 (0%) | 0 (0%) | 1 (2.1%) |  |  |
| **AVLT-recognition** |  |  |  |  |  |  |  |  |  |  |  |  |  |
| Mean (SD) | 15.1 (7.35) *** | 20.3 (4.65) c*** | 19.9 (5.01) | 13.6 (7.64) b***, d*** | 11.4 (6.65) e***,f*** |  | 12.0 (3.49) *** | 13.3 (2.12) c** | 12.6 (2.88) | 12.6 (2.03) b* | 8.43 (4.92) e***,f*** |  |  |
| Missing |  |  |  |  |  |  | 2 (0.8%) | 1 (1.2%) | 0 (0%) | 0 (0%) | 1 (2.1%) |  |  |
| **AFT** |  |  |  |  |  |  |  |  |  |  |  |  |  |
| Mean (SD) | 12.9 (5.99) *** | 17.5 (4.65) c*** | 15.5 (4.38) | 11.9 (5.37) b***, d** | 9.60 (5.12) e***,f** |  | 19.6 (5.75) *** | 21.8 (4.77) c*** | 19.6 (5.18) a* | 19.0 (5.69) b* | 16.1 (6.41) e*** |  |  |
| Missing |  |  |  |  |  |  | 1 (0.4%) | 0 (0%) | 0 (0%) | 0 (0%) | 1 (2.1%) |  |  |
| **BNT** |  |  |  |  |  |  |  |  |  |  |  |  |  |
| Mean (SD) | 19.0 (7.69) *** | 24.3 (2.97) c*** | 23.8 (3.49) | 17.4 (7.41) b***, d*** | 15.1 (8.39) e*** |  | 27.8 (3.30) * | 28.5 (2.11) | 29.0 (1.83) | 29.3 (0.866) | 25.1 (4.78) |  |  |
| Missing |  |  |  |  |  |  | 200 (82.3%) | 72 (85.7%) | 54 (88.5%) | 39 (76.5%) | 35 (74.5%) |  |  |
| **STT-A** |  |  |  |  |  |  |  |  |  |  |  |  |  |
| Mean (SD) | 183 (301) *** | 50.7 (17.0) c*** | 58.2 (23.0) | 176 (298) b***, d* | 317 (387) e***,f*** |  | 36.0 (18.8) *** | 31.0 (9.83) c*** | 34.2 (9.24) | 34.4 (15.9) | 49.2 (32.9) f* |  |  |
| Missing |  |  |  |  |  |  | 1 (0.4%) | 0 (0%) | 0 (0%) | 0 (0%) | 1 (2.1%) |  |  |
| **STT-B** |  |  |  |  |  |  |  |  |  |  |  |  |  |
| Mean (SD) | 319 (327) *** | 129 (37.3) c*** | 157 (53.9) a* | 317 (321) b***, d* | 492 (383) e***,f*** |  | 95.4 (61.8) *** | 75.1 (36.9) c*** | 94.6 (52.1) a* | 90.3 (55.0) | 141 (91.4) f* |  |  |
| Missing |  |  |  |  |  |  | 4 (1.6%) | 1 (1.2%) | 0 (0%) | 0 (0%) | 3 (6.4%) |  |  |

Note: a: CU- vs. CU+, b: CU- vs. CI-, c: CU- vs. CI+, d: CU+ vs. CI-, e: CU+ vs. CI+, f: CI- vs. CI+; *, **, and *** represent significant differences across groups at p < 0.05, 0.01, and 0.001. Values are presented as mean ± standard deviation (SD) for continuous variables and n (%) for categorical variables. P-values were calculated using ANOVA for continuous variables and χ² tests for categorical variables.

Abbreviations: CU−, cognitively unimpaired and amyloid-negative; CU+, cognitively unimpaired and amyloid-positive; CI−, cognitively impaired and amyloid-negative; CI+, cognitively impaired and amyloid-positive; SUVR, standardized uptake value ratio; MMSE, Mini-Mental State Examination. AVLT = Rey Auditory Verbal Learning Test; BNT = Boston Naming Test; AFT = Animal Fluency Test; The Chinese version of the Trail Making Test was revised to Shape Trail Test (STT).

**Supplemental Table 2. Follow-up Demographic Information.**

|  | **ADNI** |  |  |  |  |
| --- | --- | --- | --- | --- | --- |
|  | **Total (N=101)** | **CU- (N=31)** | **CU+ (N=27)** | **CI- (N=26)** | **CI+ (N=17)** |
| **age** |  |  |  |  |  |
| Mean (SD) | 76.3 (6.51) ^**^ | 75.7 (6.60) | 80.0 (5.16)^a*^ | 73.2 (6.14)^d***^ | 76.1 (6.42) |
| **Sex (%)** |  |  |  |  |  |
| Male | 48 (47.5%) | 15 (48.4%) | 10 (37.0%) | 15 (57.7%) | 8 (47.1%) |
| Female | 53 (52.5%) | 16 (51.6%) | 17 (63.0%) | 11 (42.3%) | 9 (52.9%) |
| **Education, year** |  |  |  |  |  |
| Mean (SD) | 16.4 (2.65) ^**^ | 17.2 (2.44) ^c**^ | 16.0 (2.59) | 16.9 (2.89) | 14.6 (1.93) ^f*^ |
| **APOE ε4 (%)** |  |  |  |  |  |
| non-carrier | 70 (69.3%) ^***^ | 28 (90.3%) ^c**^ | 18 (66.7%) | 20 (76.9%) | 4 (23.5%) ^f**^ |
| carrier | 31 (30.7%) | 3 (9.7%) | 9 (33.3%) | 6 (23.1%) | 13 (76.5%) |
| **Centiloids** |  |  |  |  |  |
| Mean (SD) | 30.4 (43.6) ^***^ | -1.00 (9.41) ^c***^ | 69.1 (39.2)^a***^ | -0.462 (13.7) ^d***^ | 73.6 (31.0) ^f***^ |
| **Braak I (SUVR)** |  |  |  |  |  |
| Mean (SD) | 1.17 (0.201) ^***^ | 1.10 (0.0967) ^c***^ | 1.20 (0.185) | 1.09 (0.146) | 1.39 (0.265)^e**^, ^f***^ |
| **Braak III IV (SUVR)** |  |  |  |  |  |
| Mean (SD) | 1.16 (0.148) ^***^ | 1.12 (0.0879) ^c***^ | 1.17 (0.126) | 1.09 (0.0872) | 1.31 (0.222) ^e**, f**^ |
| **Braak V VI (SUVR)** |  |  |  |  |  |
| Mean (SD) | 1.06 (0.123) ^***^ | 1.04 (0.0860) ^c**^ | 1.08 (0.100) | 1.01 (0.0756) | 1.17 (0.193) ^f***^ |
| **MMSE** |  |  |  |  |  |
| Mean (SD) | 28.2 (2.64) ^***^ | 29.3 (0.864) ^c***^ | 28.0 (2.24) | 28.8 (1.62) | 25.3 (4.24) ^e**, f***^ |
| **APOE genotype** |  |  |  |  |  |
| E2/E3 | 8 (7.9%) | 2 (6.5%) | 4 (14.8%) | 2 (7.7%) | 0 (0%) |
| E2/E4 | 2 (2.0%) | 0 (0%) | 0 (0%) | 0 (0%) | 2 (11.8%) |
| E3/E3 | 62 (61.4%) | 26 (83.9%) | 14 (51.9%) | 18 (69.2%) | 4 (23.5%) |
| E3/E4 | 23 (22.8%) | 2 (6.5%) | 8 (29.6%) | 6 (23.1%) | 7 (41.2%) |
| E4/E4 | 6 (5.9%) | 1 (3.2%) | 1 (3.7%) | 0 (0%) | 4 (23.5%) |
| **GFAP (pg/mL)** |  |  |  |  |  |
| Mean (SD) | 169 (78.5) ^***^ | 151 (63.3) ^c*^ | 206 (90.0) ^a*^ | 122 (40.9) ^d***^ | 213 (81.8) ^f***^ |
| **pTau181 (pg/mL)** |  |  |  |  |  |
| Mean (SD) | 21.7 (9.79) ^***^ | 18.1 (8.09) | 26.9 (11.8) ^a**^ | 18.6 (7.43) ^d**^ | 24.7 (8.23) |
| **pTau217 (pg/mL)** |  |  |  |  |  |
| Mean (SD) | 0.474 (0.317) ^***^ | 0.303 (0.135) ^c***^ | 0.745 (0.372) ^a***^ | 0.299 (0.139) ^d***^ | 0.664 (0.265) ^f***^ |
| Missing | 9 (8.9%) | 2 (6.5%) | 1 (3.7%) | 1 (3.8%) | 5 (29.4%) |
| **AVLT_LDR** |  |  |  |  |  |
| Mean (SD) | 10.3 (3.40) ^***^ | 12.1 (2.31) ^c***^ | 10.5 (3.07) | 10.3 (3.53) | 6.41 (2.32) ^e***, f***^ |
| **AVLT-recognition** |  |  |  |  |  |
| Mean (SD) | 12.2 (3.12) ^***^ | 13.8 (1.36) ^c***^ | 12.8 (2.60) | 12.3 (2.05) | 8.29 (4.27) ^e***, f***^ |
| **AFT** |  |  |  |  |  |
| Mean (SD) | 20.5 (5.86) ^***^ | 22.7 (5.03) ^c***^ | 21.0 (5.02) | 20.9 (6.47) | 14.9 (4.12) ^e**, f**^ |
| **BNT** |  |  |  |  |  |
| Mean (SD) | 28.1 (2.52) ^*^ | 28.0 (2.61) | 28.5 (2.38) | 29.6 (0.726) | 25.2 (2.68) ^f**^ |
| Missing | 77 (76.2%) | 25 (80.6%) | 23 (85.2%) | 17 (65.4%) | 12 (70.6%) |
| **STT-A** |  |  |  |  |  |
| Mean (SD) | 34.1 (16.3) ^**^ | 28.9 (9.15) ^c**^ | 33.4 (8.87) | 33.1 (16.4) | 46.2 (27.3) ^e*, f*^ |
| **STT-B** |  |  |  |  |  |
| Mean (SD) | 90.0 (64.9) ^***^ | 60.3 (27.1) ^c***^ | 85.6 (42.7) | 85.1 (59.8) | 159 (98.2) ^e***, f***^ |

Note: a: CU- vs. CU+, b: CU- vs. CI-, c: CU- vs. CI+, d: CU+ vs. CI-, e: CU+ vs. CI+, f: CI- vs. CI+; *, **, and *** represent significant differences across groups at p < 0.05, 0.01, and 0.001. Values are presented as mean ± standard deviation (SD) for continuous variables and n (%) for categorical variables. P-values were calculated using ANOVA for continuous variables and χ² tests for categorical variables.

Abbreviations: CU−, cognitively unimpaired and amyloid-negative; CU+, cognitively unimpaired and amyloid-positive; CI−, cognitively impaired and amyloid-negative; CI+, cognitively impaired and amyloid-positive; SUVR, standardized uptake value ratio; MMSE, Mini-Mental State Examination. AVLT = Rey Auditory Verbal Learning Test; BNT = Boston Naming Test; AFT = Animal Fluency Test; The Chinese version of the Trail Making Test was revised to Shape Trail Test (STT).


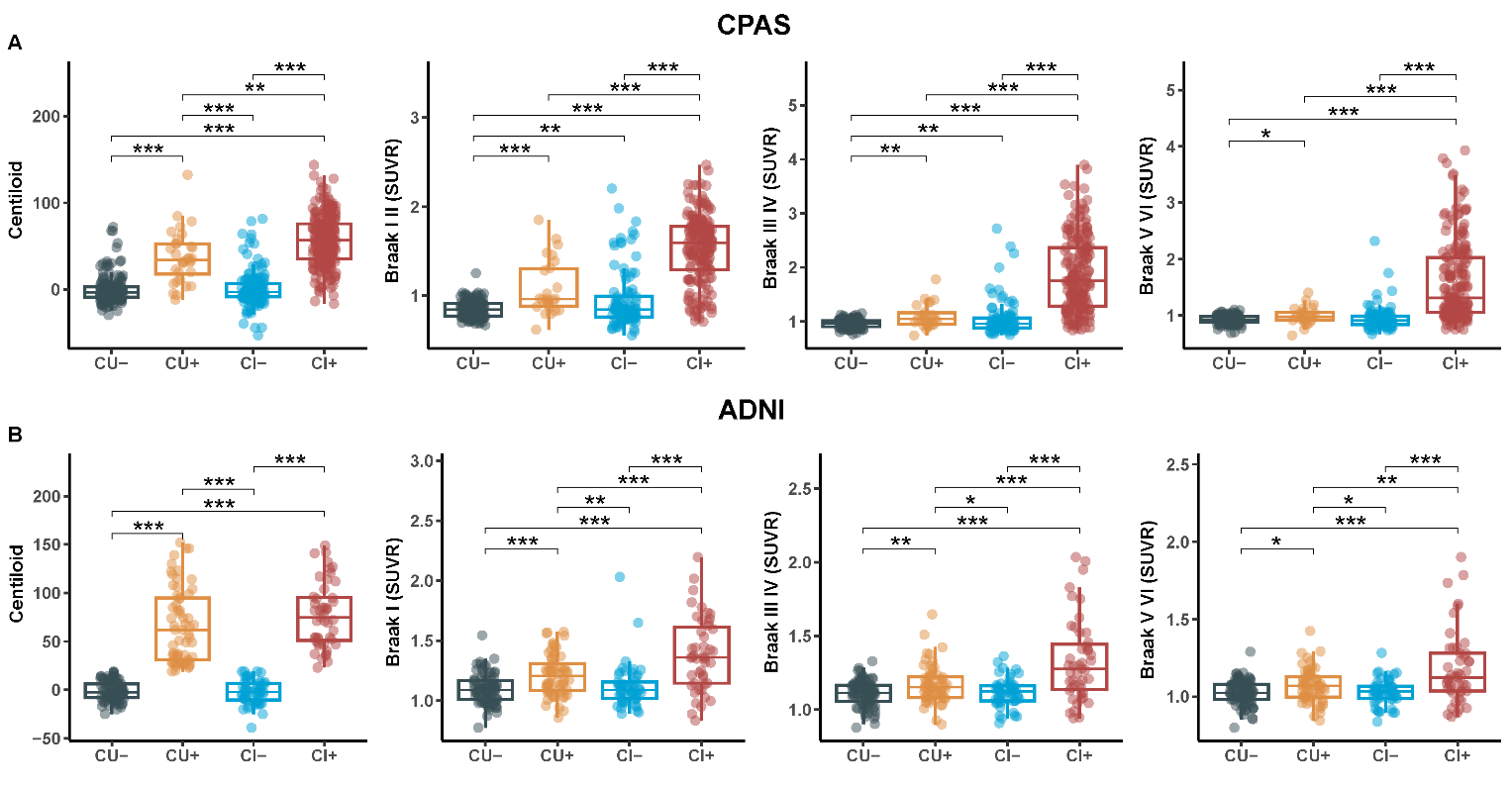
**Supplemental Fig. 1 Aβ PET (Centiloid) and Tau PET (SUVR) across diagnostic groups stratified by Aβ status, adjusted for age and sex.** Comparisons across Aβ status groups: cognitively unimpaired with negative Aβ (CU-), cognitively unimpaired with positive Aβ (CU+), cognitively impaired with negative Aβ (CI-), and cognitively impaired with positive Aβ (CI+). Significant differences between groups are indicated by *p < 0.05, **p < 0.01, ***p < 0.001 (Bonferroni-corrected for multiple comparisons).

**
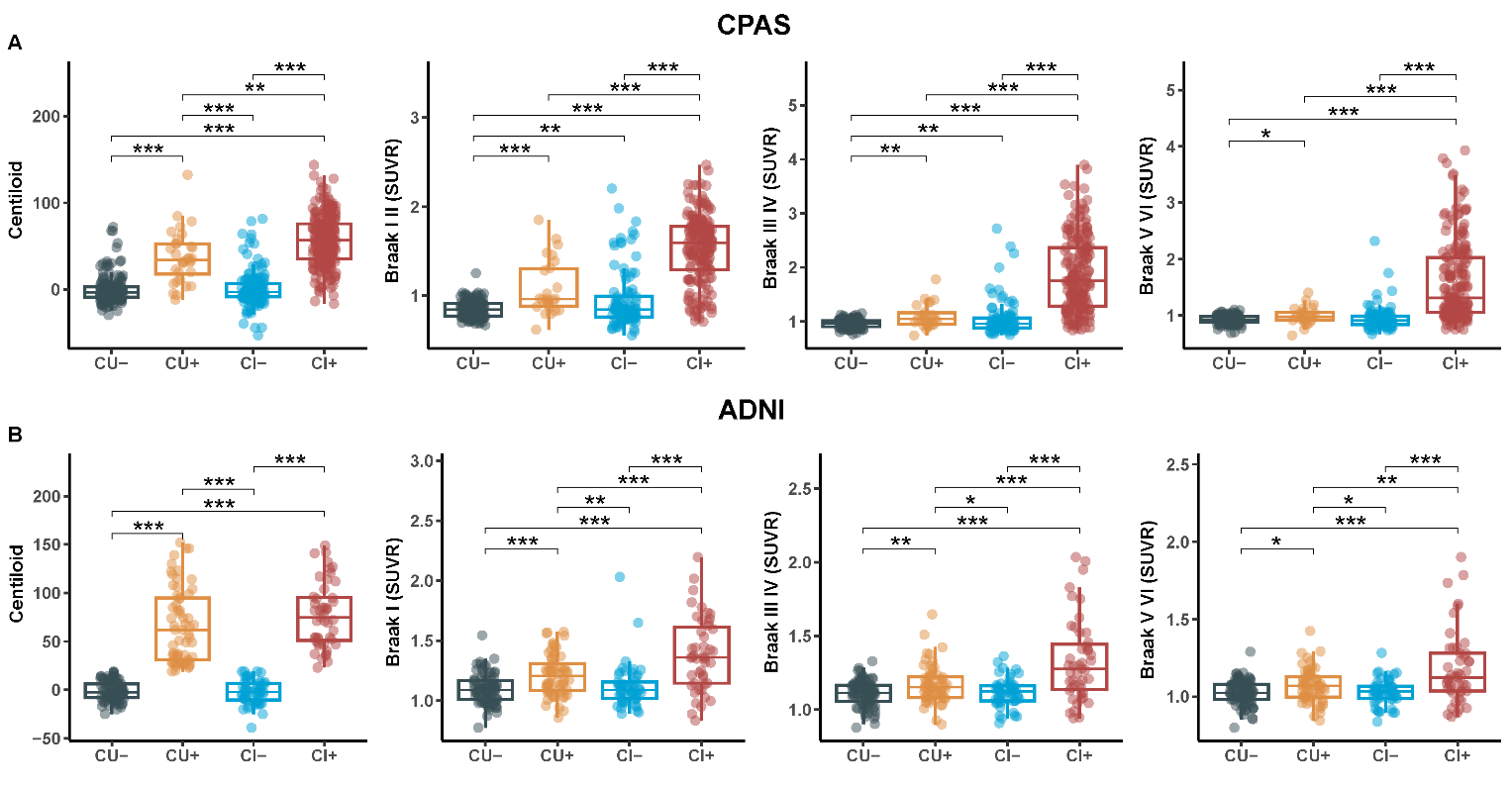
**

**Supplemental Fig. 2** **Plasma biomarker levels across diagnostic groups stratified by Aβ and APOE ε4 status, adjusted for age and sex.** All plasma biomarker values were log-transformed prior to analysis. (A, C) Comparisons across Aβ status groups: cognitively unimpaired with negative Aβ (CU-), cognitively unimpaired with positive Aβ (CU+), cognitively impaired with negative Aβ (CI-), and cognitively impaired with positive Aβ (CI+). (B, D) Comparisons across APOE ε4 carrier status groups: cognitively unimpaired non-carriers (CU-nonε4), cognitively unimpaired carriers (CU-ε4), cognitively impaired non-carriers (CI-nonε4), and cognitively impaired carriers (CI-ε4). Significant differences
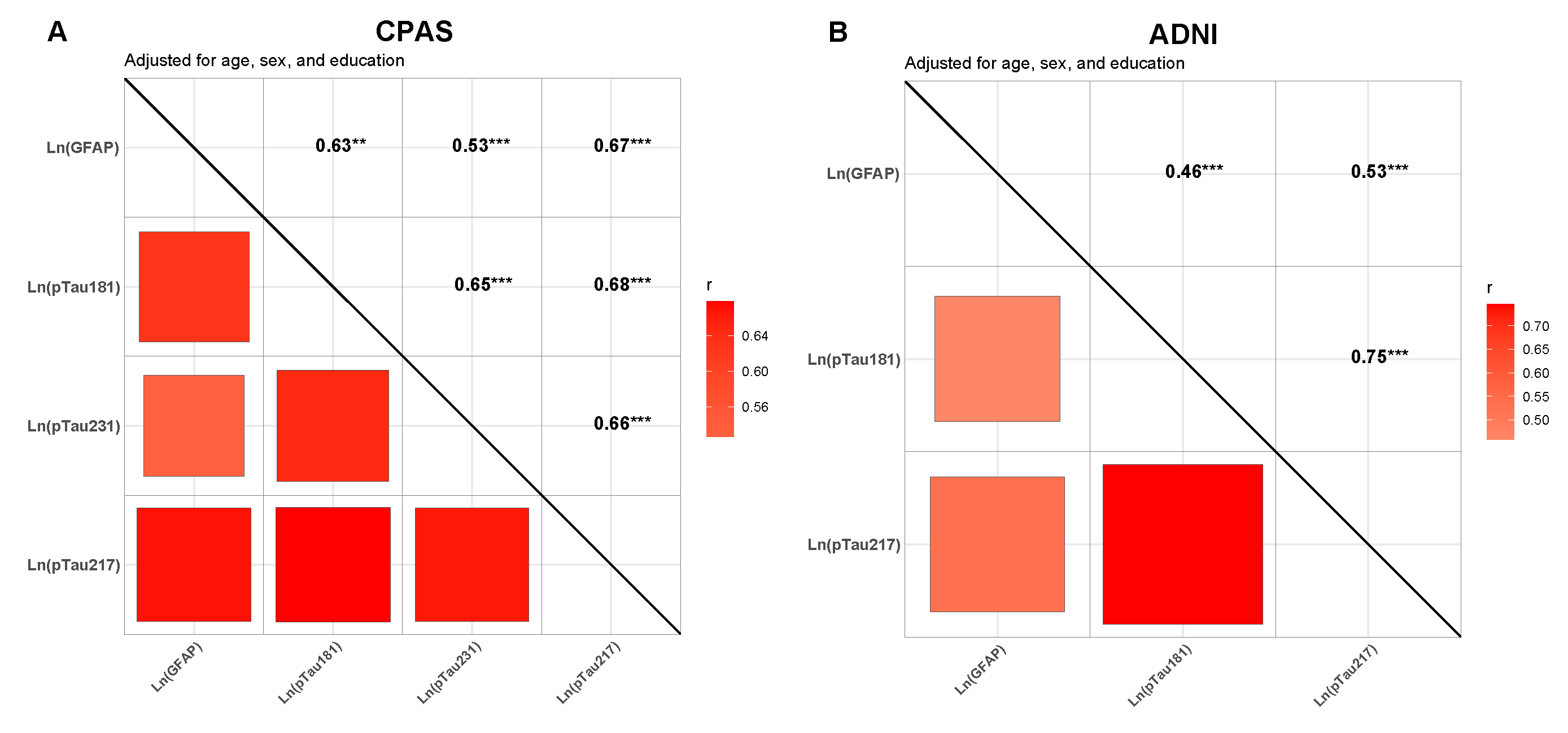
between groups are indicated by *p < 0.05, **p < 0.01, ***p < 0.001 (Bonferroni-corrected for multiple comparisons).


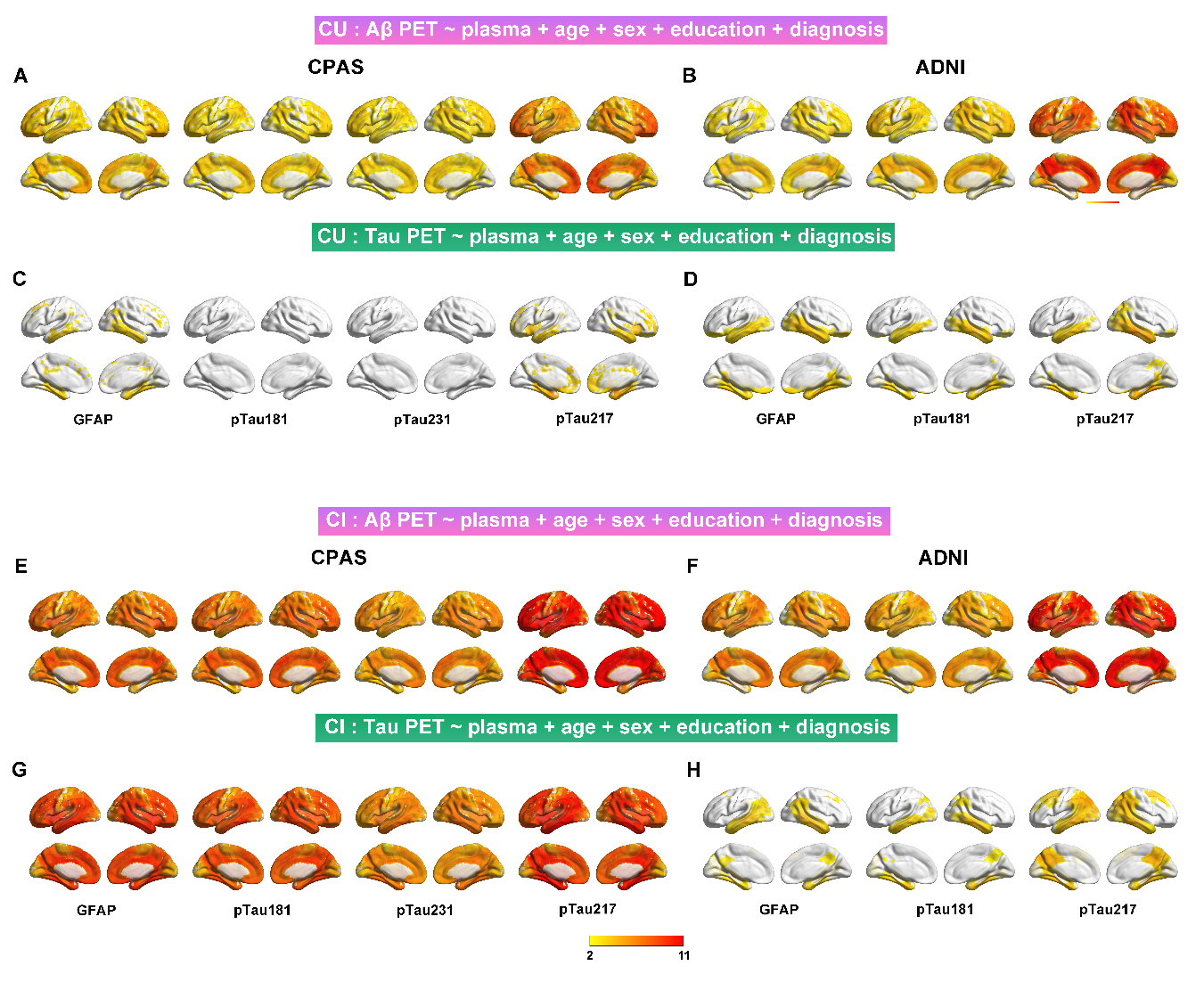
**Supplemental Fig. 3 Associations between plasma biomarkers.** All plasma biomarker values were log-transformed prior to analysis. The statistical model used is Partial correlation, with r values adjusted for age, sex, education. *p < 0.05, **p < 0.01, ***p < 0.001

**Supplemental Fig. 4 Associations of plasma GFAP and phosphorylated tau with Aβ and Tau PET in cognitively unimpaired (CU) and cognitively impaired (CI) diagnostic groups.** Voxel-wise associations between plasma GFAP and pTau with Aβ PET were evaluated in the CPAS (A) and ADNI (B) cohorts, and with Tau PET in CPAS (C) and ADNI (D) among CU participants. Corresponding analyses in CI participants are shown for associations with Aβ PET in CPAS (E) and ADNI (F), and with Tau PET in CPAS (G) and ADNI (H). Voxel-wise statistical maps display T-values, with significance defined at p < 0.05 (peak-level FDR correction). All models were adjusted for age, sex, and years of education. Plasma GFAP and pTau values were log-transformed prior to inclusion in the models.


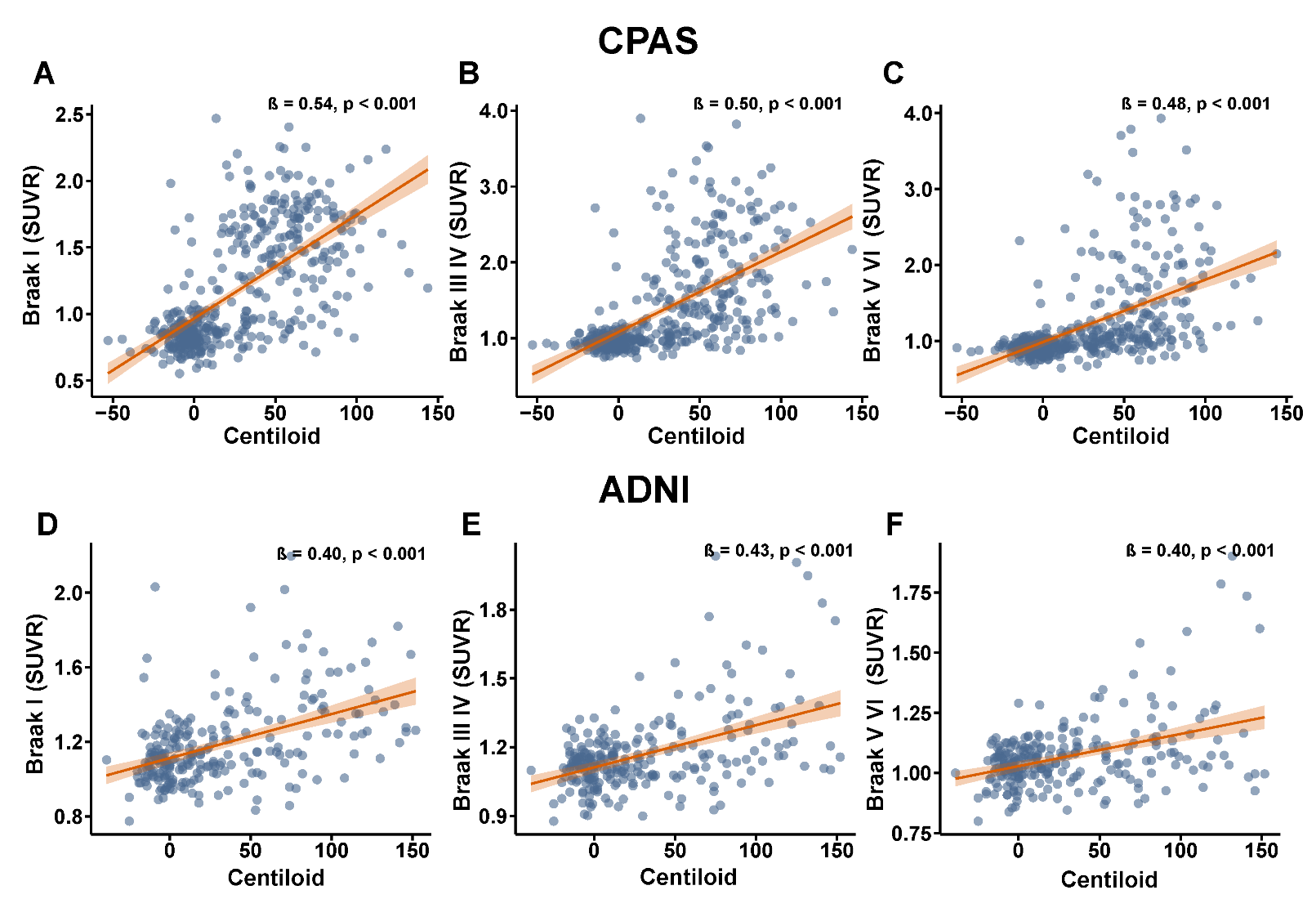
**Supplemental Fig. 5** **Association between Aβ PET (Centiloid) and tau PET (SUVR).** Linear regression analysis adjusted for age, sex, years of education, and diagnosis.

**
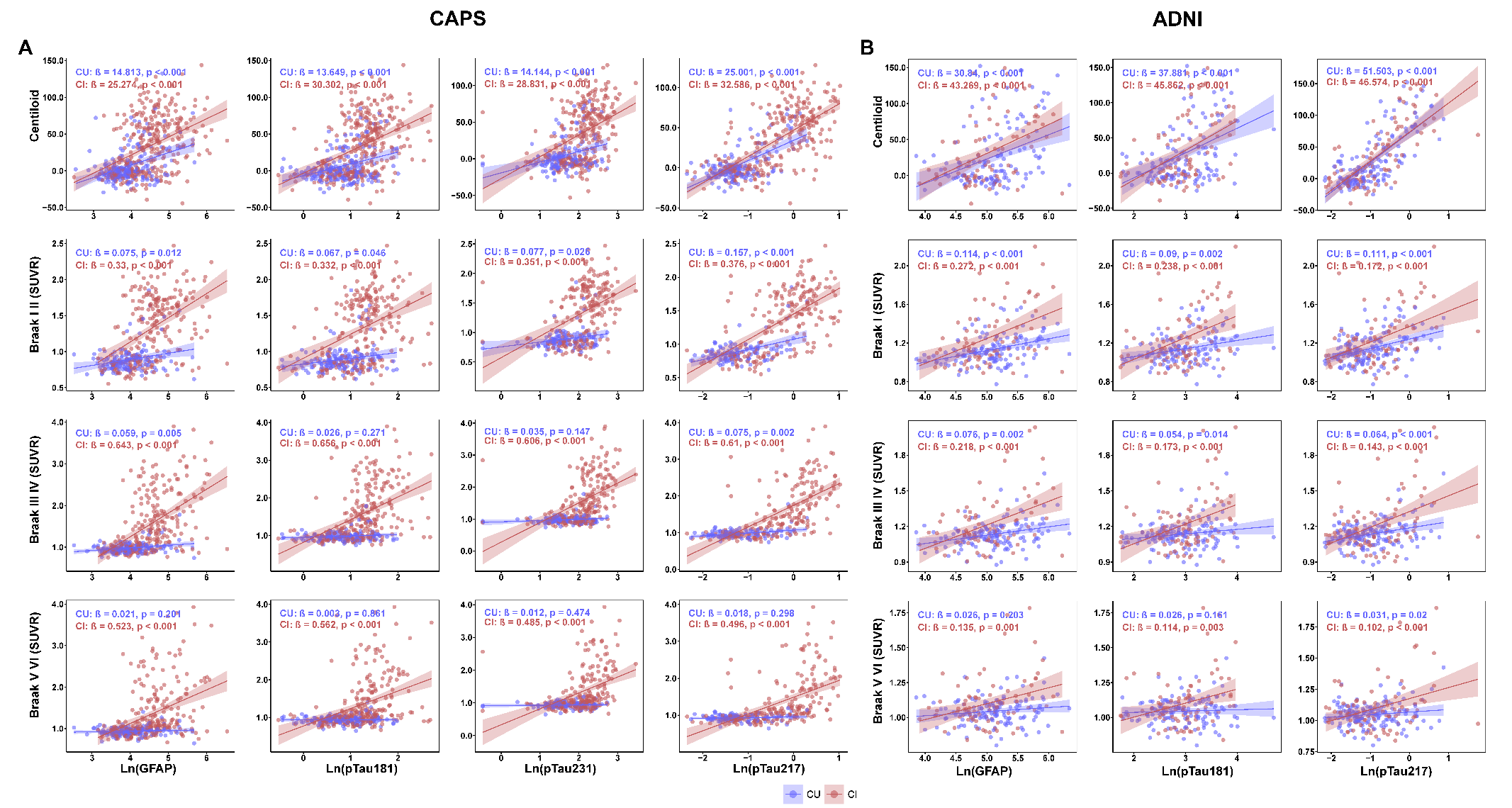
****Supplemental Fig. 6 Associations of plasma phosphorylated tau (pTau) with Aβ and Tau PET based on diagnostic groups.** Plasma pTau were assessed for their associations with Aβ PET and Tau PET based on diagnostic groups at the ROI level in the CPAS (A) and ADNI (B) cohorts. Linear regression models are shown with 95% confidence intervals and accompanied by standardized β coefficients, stratified by diagnostic groups (red = CI, blue = CU). All models were adjusted for age, sex, years of education, clinical diagnosis and centiloid. Plasma GFAP and pTau were log-transformed prior to inclusion in the models. SUVR = standardized uptake value ratio.


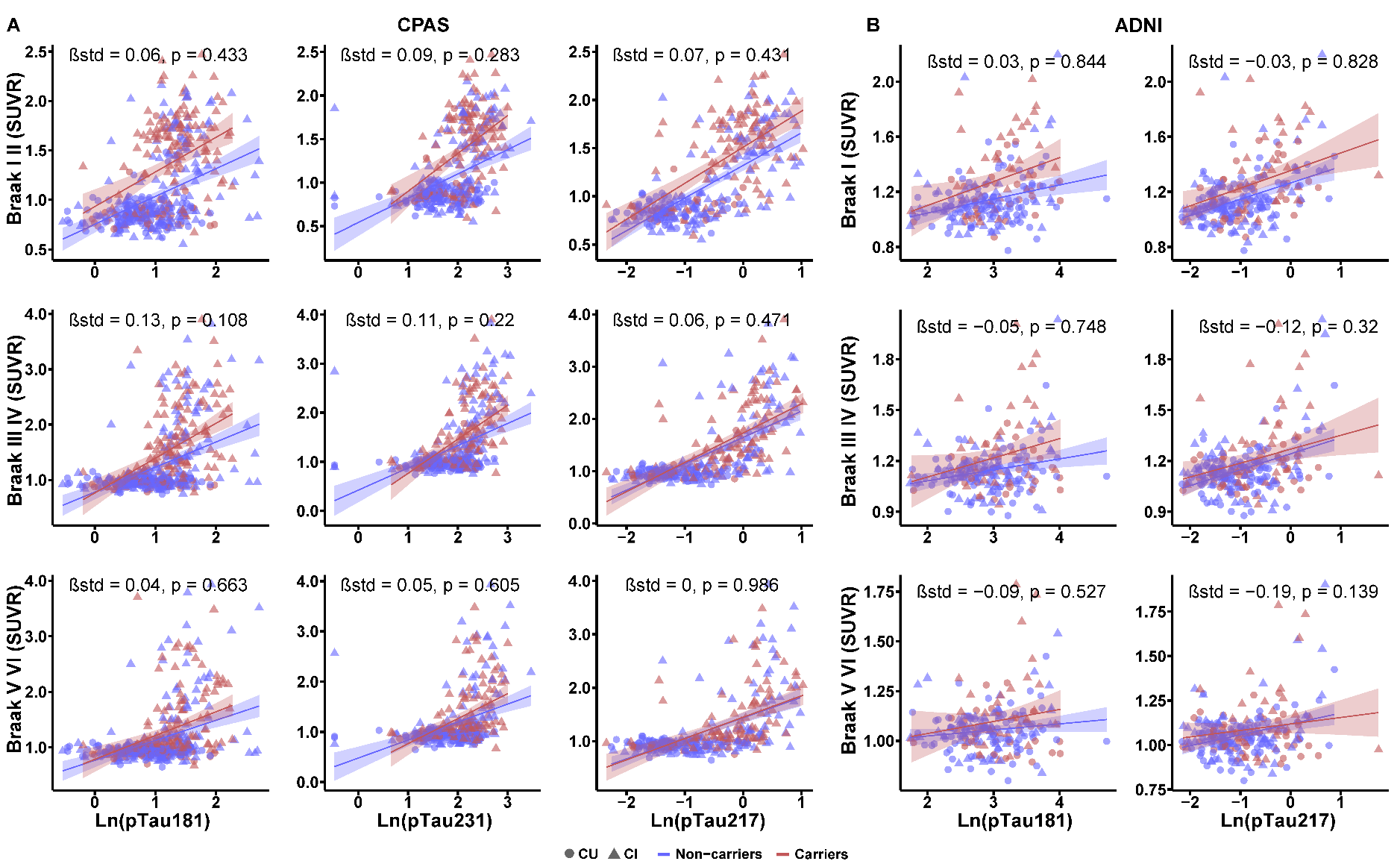
**Supplemental Fig. 7 Associations of plasma phosphorylated tau (pTau) and its interaction with APOE ε4 status with Aβ and Tau PET.** Plasma pTau and its interaction with APOE ε4 carrier status were assessed for their associations with Aβ PET and Tau PET at the ROI level in the CPAS (A) and ADNI (B) cohorts. Linear regression models are shown with 95% confidence intervals and accompanied by standardized β coefficients, along with regression lines and individual data points stratified by APOE ε4 status (red = carriers, blue = non-carriers). Circles and triangles represent CU and CI participants, respectively. All models were adjusted for age, sex, years of education, clinical diagnosis and centiloid. Plasma GFAP and pTau were log-transformed prior to inclusion in the models. SUVR = standardized uptake value ratio.


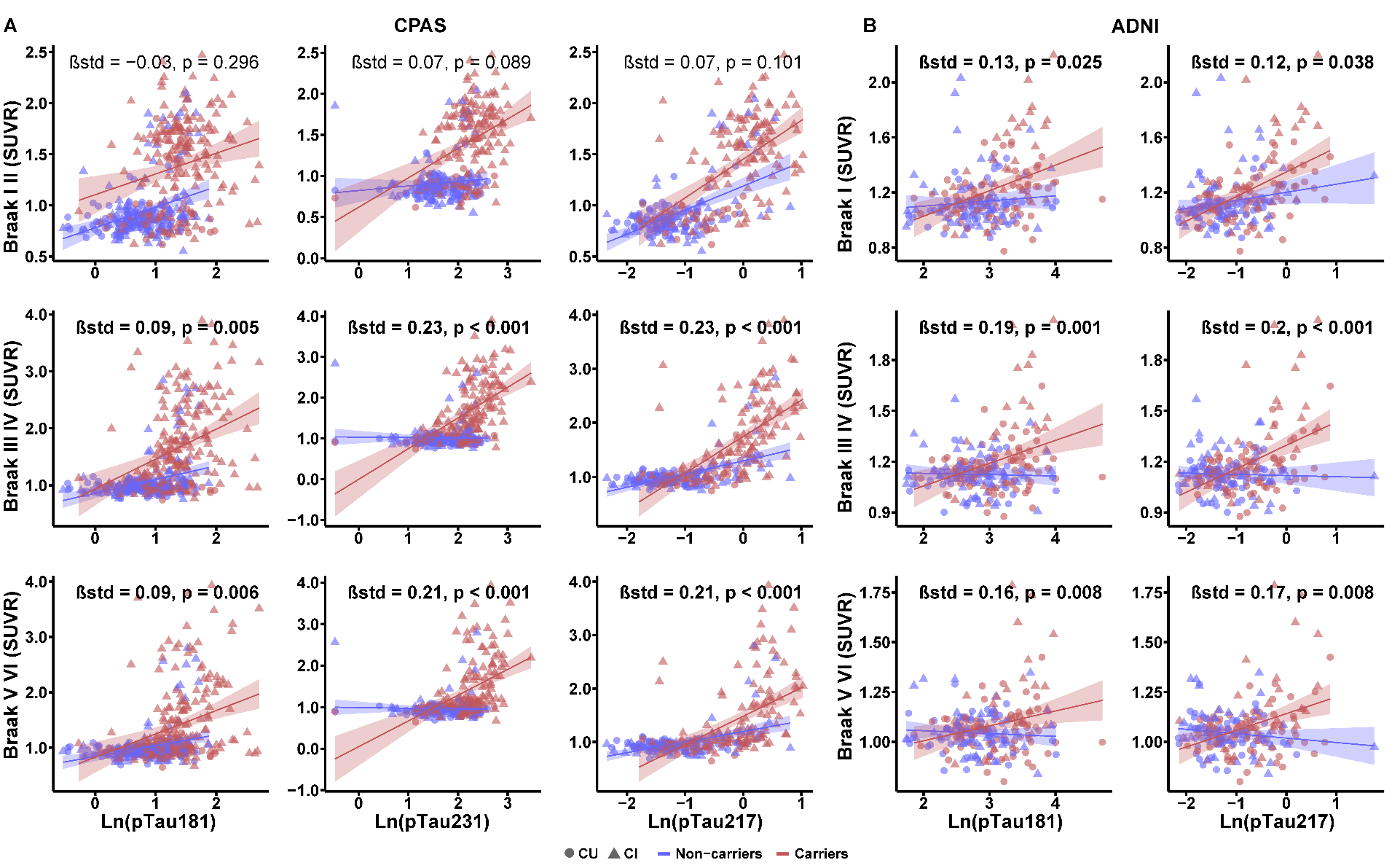
**Supplemental Fig. 8 Associations of plasma phosphorylated tau (pTau) and its interaction with GFAP with Aβ and tau PET.** Plasma pTau and its interaction with plasma GFAP were assessed for their associations with Aβ PET and Tau PET at the ROI level in the CPAS (A) and ADNI (B) cohorts. Linear regression models are shown with 95% confidence intervals and accompanied by standardized β coefficients, along with regression lines and individual data points stratified by GFAP level (red = high GFAP, blue = low GFAP). Circles and triangles represent CU and CI participants, respectively. All models were adjusted for age, sex, years of education, clinical diagnosis and centiloid. Plasma GFAP and pTau were log-transformed prior to inclusion in the models. All interactions were modeled using continuous plasma GFAP values; GFAP levels were dichotomized at the median for visualization purposes. SUVR = standardized uptake value ratio.
